# Genome-wide mapping of histone H3 lysine 4 trimethylation in *Eucalyptus grandis* developing xylem

**DOI:** 10.1186/s12870-015-0499-0

**Published:** 2015-05-10

**Authors:** Steven G Hussey, Eshchar Mizrachi, Andrew Groover, Dave K Berger, Alexander A Myburg

**Affiliations:** Department of Genetics, Forestry and Agricultural Biotechnology Institute (FABI), Genomics Research Institute (GRI), University of Pretoria, Private Bag X20, Pretoria, 0028 South Africa; US Forest Service, Pacific Southwest Research Station, Davis, CA USA; Department of Plant Biology, University of California, Davis, USA; Department of Plant Science, Forestry and Agricultural Biotechnology Institute (FABI), Genomics Research Institute (GRI), University of Pretoria, Private Bag X20, Pretoria, 0028 South Africa

**Keywords:** ChIP-seq, H3K4me3, Histone, Secondary cell wall, Xylogenesis, *Eucalyptus*

## Abstract

**Background:**

Histone modifications play an integral role in plant development, but have been poorly studied in woody plants. Investigating chromatin organization in wood-forming tissue and its role in regulating gene expression allows us to understand the mechanisms underlying cellular differentiation during xylogenesis (wood formation) and identify novel functional regions in plant genomes. However, woody tissue poses unique challenges for using high-throughput chromatin immunoprecipitation (ChIP) techniques for studying genome-wide histone modifications *in vivo*. We investigated the role of the modified histone H3K4me3 (trimethylated lysine 4 of histone H3) in gene expression during the early stages of wood formation using ChIP-seq in *Eucalyptus grandis*, a woody biomass model.

**Results:**

Plant chromatin fixation and isolation protocols were optimized for developing xylem tissue collected from field-grown *E. grandis* trees. A “nano-ChIP-seq” procedure was employed for ChIP DNA amplification. Over 9 million H3K4me3 ChIP-seq and 18 million control paired-end reads were mapped to the *E. grandis* reference genome for peak-calling using Model-based Analysis of ChIP-Seq. The 12,177 significant H3K4me3 peaks identified covered ~1.5% of the genome and overlapped some 9,623 protein-coding genes and 38 noncoding RNAs. H3K4me3 library coverage, peaking ~600 - 700 bp downstream of the transcription start site, was highly correlated with gene expression levels measured with RNA-seq. Overall, H3K4me3-enriched genes tended to be less tissue-specific than unenriched genes and were overrepresented for general cellular metabolism and development gene ontology terms. Relative expression of H3K4me3-enriched genes in developing secondary xylem was higher than unenriched genes, however, and highly expressed secondary cell wall-related genes were enriched for H3K4me3 as validated using ChIP-qPCR.

**Conclusions:**

In this first genome-wide analysis of a modified histone in a woody tissue, we optimized a ChIP-seq procedure suitable for field-collected samples. In developing *E. grandis* xylem, H3K4me3 enrichment is an indicator of active transcription, consistent with its known role in sustaining pre-initiation complex formation in yeast. The H3K4me3 ChIP-seq data from this study paves the way to understanding the chromatin landscape and epigenomic architecture of xylogenesis in plants, and complements RNA-seq evidence of gene expression for the future improvement of the *E. grandis* genome annotation.

**Electronic supplementary material:**

The online version of this article (doi:10.1186/s12870-015-0499-0) contains supplementary material, which is available to authorized users.

## Background

A rich diversity of histone modifications affect chromatin structure and/or gene activation and repression in eukaryotes reviewed by [1,2]. Chromatin organization plays a crucial role in plant gene regulation, employing conserved and unique mechanisms compared to those of other eukaryotes [3]. In mammals, as well as plants [4,5], the presence of activating histone modifications such as trimethylated lysine 4 of histone H3 (H3K4me3) and acetylated lysine 9 (H3K9Ac) at the transcription start site (TSS) are good predictors of gene expression [6]. For example, the degree of H3K4 trimethylation at the TSS is directly proportional to transcript expression level [7,8]. In mammals, monomethylated H3K4 (H3K4me1) is preferentially associated with enhancer elements, while dimethylated H3K4 (H3K4me2) is associated with enhancers and promoters, as well as with “poised” genes that are expressed at defined developmental stages or in specific cell types [7,9]. H3K36 methylation, in contrast, is thought to mediate RNA polymerase II (Pol II) elongation and act as docking sites for transcript-processing enzymes reviewed by [10]. In general, plants have a similar histone code to that of mammals, with some exceptions such as a higher abundance of H3K4me2 reviewed by [11].

Lysine 4 of histone H3 is trimethylated by SET1 of the *Trithorax* protein complex COMPASS in yeast [12], with ATXR3 and to some extent ATX1 performing this function in *Arabidopsis* [13-16]. In yeast, H3K4 trimethylation is predicated on Rad6-mediated ubiquitination of lysine 123 of histone H2B (uH2B-K123) [17,18]. The uH2B-K123 modification is critical for H3K4 methylation by SET1, possibly acting to open the chromatin structure for SET1 targeting [18]. SET1 associates with the activated form of Pol II, in part through the PAF1 complex, ensuring that H2B ubiquitination and H3K4 methylation occur proximal to the pre-initiation complex reviewed by [19]. Thus, H3K4me3 appears to be established by active transcription itself, is reported to occur at over 90% of Pol II-enriched sites in human [8] and is associated with transcription initiation but not necessarily transcription elongation in mammals [20]. Since the H3K4me3 modification endures at previously active genes for up to several hours after silencing in yeast, it represents evidence of both active and recent transcription [21]. H3K4 methylation can, however, be dynamically reversed by histone demethylases [11,22]. The function of H3K4me3 is to recruit TFIID to active promoters and assisting in pre-initiation complex formation, which is enhanced in the presence of a TATA box [23], via interaction with the TAF3 subunit [24,25]. A number of other proteins are known to bind to H3K4me3 at specific loci, which are in turn tethered to, or recruit, enzymes that manipulate the local chromatin structure [2].

At human TSSs, “open” chromatin regions that are hypersensitive to DNase I cleavage are followed by a prominent H3K4me3 signal immediately downstream; a relationship so strong that the pattern can be used to annotate TSSs and the direction of transcription [26]. In plants, H3K4me3 histone modifications occur almost exclusively in genes and their promoters but preferentially occupy genic regions 250–600 bp (*Arabidopsis*) or 500–1000 bp (*Oryza*) downstream of the TSS [27-30]. Genes occupied by H3K4me3, especially in the absence of H3K4me1 and H3K4me2, generally display low tissue specificity but high levels of constitutive expression in *Arabidopsis* [27,28]. However in two drought studies, H3K4me3 distribution broadened considerably along genes differentially expressed during drought stress in *Arabidopsis* [29], and showed differential trimethylation for a proportion of genes differentially expressed during drought stress in rice [31], suggesting H3K4me3 can also be associated with tightly regulated pathways.

Due to the widespread use of woody biomass in pulp, paper and chemical cellulose industries, various studies have undertaken to understand the transcriptional regulation of xylogenesis (wood formation) [32-34]. Modified histones have been poorly studied in woody tissues, despite their importance in growth and development. Secondary xylem, which forms the characteristic swelling of woody plant stems, develops from xylem mother cells in the vascular cambium, a lateral meristem [35]. Xylem mother cells form nascent fusiform initials that give rise to fibers and vessels, the two main cell types constituting secondary xylem, undergoing elongation, secondary cell wall deposition, lignification and programmed cell death within a thin layer of tissue (650–1000 μm in *Populus* [36]) known as developing secondary xylem (DSX) [37,38]. Chromatin immunoprecipitation (ChIP) has only recently been applied to vascular tissues to study protein-DNA interactions [39,40]. These have been restricted to the DSX tissue rather than mature xylem, since dead or dying cells and large quantities of secondary cell wall material characterising fibers and vessels pose significant challenges to nuclei isolation.

Here, we aimed to determine the role of the activating histone modification H3K4me3 in the epigenomic regulation of xylogenesis, using field-growing *Eucalyptus grandis* trees as our model. We hypothesized that H3K4me3 signals marking Pol II-transcribed genes, including those involved in wood formation, can predict their corresponding transcript levels in developing xylem. We assessed and optimized existing protocols for the isolation of crosslinked chromatin from field-collected DSX tissue for use in ChIP-seq assays, and modified a nano-ChIP-seq protocol for the amplification of ChIP DNA. To the best of our knowledge, this is the first genome-wide study of the role of a modified histone in developing wood.

## Results

### ChIP-seq analysis of H3K4me3 in *E. grandis* developing secondary xylem

We collected DSX samples in spring from two seven-year-old *E. grandis* individuals (clonal ramets) growing in a plantation. We optimized chromatin fixation, isolation and sonication and assessed isolated chromatin quality using micrococcal nuclease (see Additional file 1: Supplementary Note S1 and Additional file 2: Figures S1-S4). We then conducted a ChIP-seq analysis of the activating histone mark H3K4me3 to evaluate our modified ChIP-seq protocol (see Methods) and to better understand the role of this signature in developing xylem gene regulation. We selected a commercial antibody for H3K4me3 which had been validated for ChIP analyses in *Arabidopsis* [15,41-43]. Antibody recognition of the H3Kme3 protein in *Eucalyptus* DSX was confirmed by Western blot analysis of DSX nuclear extracts, where the antibody recognized a ~17 kDa band corresponding to the predicted molecular weight of H3K4me3 (Additional file 2: Figure S5a). Additionally, a dotblot assay using synthetic peptides representing all possible methylated and non-methylated variants of H3K4me3 showed that the antibody specifically recognized only the trimethylated variant (Additional file 2: Figure S5b). In trial experiments, different amounts of anti-H3K4me3 antibody produced similar enrichments of candidate regions as assessed by ChIP-qPCR (Additional file 2: Figure S6).

We generally obtained 1–2 ng ChIP-enriched DNA from the modified protocol by Kaufmann *et al.* [44]. In order to perform Illumina sequencing with enough ChIP DNA to spare for qPCR validation, we adopted a “nano-ChIP-seq” approach developed for ChIP-seq analysis of limited mammalian cell numbers [45,46]. Modifications to the Adli & Bernstein [45] ChIP DNA amplification protocol (see Methods) allowed for successful amplification of 1 ng or less of template (Additional file 2: Figure S7), producing up to several hundred nanograms of template for library preparation.

Following ChIP DNA amplification and library construction, we generated over 30 million 50-base paired-end reads from both the H3K4me3-enriched and input (control) libraries (Additional file 3: Table S1). The sequences were trimmed to remove primer sequences and mapped to the v.1.1. annotation of the *E. grandis* reference genome [47]. For one individual (V11), we additionally sequenced an IgG_2a_ negative control library to remove false positive peaks due to nonspecific antibody or protein A binding (see Methods). Between 3.7 and 11.7 million read pairs mapped uniquely for each H3K4me3 and input replicate after filtering for PCR-induced duplicated reads (Additional file 3: Table S1). Only 9.8% of IgG_2a_ library reads mapped to the genome, likely reflecting the lower complexity of non-specifically bound targets. Read coverage along the genome correlated significantly (*r* = 0.90, *P* < 2.2 × 10^−16^) between biological replicates (Additional file 2: Figure S8). Strand cross-correlation analysis showed that all H3K4me3 ChIP libraries were enriched to an efficiency well within ENCODE standards [48] (Additional file 2: Figure S9).

We followed ENCODE guidelines [49] for peak-calling using Model-based Analysis of ChIP-seq (MACS v.2.0) software [50], employing the Irreproducible Discovery Rate (IDR) method to identify peaks from ChIP-seq data from both replicates with a low false positive rate (IDR < 0.01) and a high degree of biological replication (IDR < 0.05; see Methods). To assess within- and between-sample consistency, the number of shared peaks identified separately for biological replicates, randomly generated within-sample pseudoreplicates, and randomly generated pseudoreplicates of pooled data were within two-fold of each other (Additional file 3: Table S2), in agreement with ENCODE recommendations [48]. Within-sample pseudoreplicates of each biological replicate produced similar IDR profiles (Additional file 2: Figure S10), indicating similar data quality for each biological replicate.

After removing 261 false positive peaks shared with the IgG_2a_ negative control sample, our method identified 12,177 significant H3K4me3 peaks (Additional file 4). Subsampling of increasing proportions of the mapped tags showed that the number of peaks called separately for each replicate began to plateau (Additional file 2: Figure S11), suggesting near-saturation of H3K4me3 peak detection at the sequencing depths obtained in this study. The peaks, which spanned a median interval of 781 bp (Additional file 2: Figure S12), covered 10.14 Mb (~1.5%) of the assembled genome, ~86% of which overlapped annotated gene models and/or promoter regions within 1 kb upstream of the predicted TSS. 9,623 target genes were identified as enriched for H3K4me3 based on their physical overlap with a significant peak (Additional file 5). Of the 2,043 peaks that did not overlap a gene model in the v.1.1. genome annotation, a further 196 overlapped some 186 low-confidence gene annotations that were previously removed from the first annotation (i.e. v.1.0), suggesting that some of these may be *bona fide* gene models (Additional file 6).

**Figure 1:**
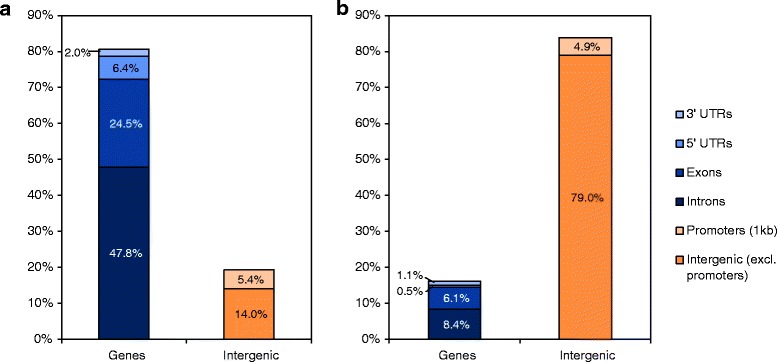
Overlap of H3K4me3 ChIP-seq peaks with genomic features. **(a)** Percentage of all H3K4me3 peak intervals overlapping different genomic features. The nonredundant set of annotated exons, introns, 5’ untranslated regions (5’ UTRs) and 3’ untranslated regions (3’ UTRs) has been collated as representing genes, whereas 1 kb upstream promoter regions and the remaining genomic regions are classified as “intergenic”. **(b)** Percentage of the *E. grandis* genome assigned to different genomic features according to the v.1.1 annotation.

On average, ~48% of a given peak interval, defined here as the genomic span of a significant peak, overlapped intronic sequence, and ~25% overlapped exon sequence (Figure 1a). In intergenic regions, ~5% of peak intervals overlapped 1 kb promoter regions of genes (Figure 1a). Thus, compared to the genomic frequency of these annotations (Figure 1b), H3K4me3 peak distribution was heavily biased towards genes. We also assessed the H3K4me3 enrichment of known and predicted noncoding RNA (ncRNA) elements in the *E. grandis* genome [51]. Disregarding ambiguous H3K4me3 peaks that overlapped with both ncRNAs and genes, ~18% of small nucleolar RNAs (snoRNAs) and ~2% of known or predicted microRNAs (miRNAs) were enriched for H3K4me3 whereas transfer RNAs (tRNAs), ribosomal RNAs (rRNAs), small nuclear RNAs (snRNAs), antisense RNAs and small RNAs (sRNAs) showed no or significantly less enrichment (Table 1). A number of putative targets of the enriched miRNAs were identified that remain to be experimentally verified (Additional file 3: Table S3). The enriched snoRNAs appeared to consist of 14 polycistronic clusters (not shown), a common arrangement in plants [52]. These data are consistent with the fact that miRNAs and many snoRNAs are transcribed by Pol II and might hence be expected to exhibit H3K4me3 modifications when expressed [53,54].

**Table 1:** **ncRNA elements enriched for H3K4me3**

**ncRNA class**	**H3K4me3-enriched** ^**a**^	**Total annotations**	**% enriched** ^**a**^
Predicted snoRNAs	31 (59)	175	17.7 (33.7)
Predicted miRNAs	3 (4)	153	2.0 (2.6)
Known miRNAs	1 (2)	60	1.7 (3.3)
Predicted tRNAs	3 (8)	508	0.6 (1.57)
Predicted antisense RNAs	0 (0)	19	0.0 (0.0)
Predicted rRNAs	0 (0)	269	0.0 (0.0)
Predicted spliceosomal snRNA	0 (0)	125	0.0 (0.0)
Predicted sRNAs	0 (0)	80	0.0 (0.0)

^a^Excludes H3K4me3 peaks overlapping with annotated protein-coding genes. ncRNAs overlapping peaks that also overlap genes are indicated in parenthesis.

Putative targets of H3K4me3-enriched miRNAs are indicated in Additional file 3: Table S3.

**Figure 2:**
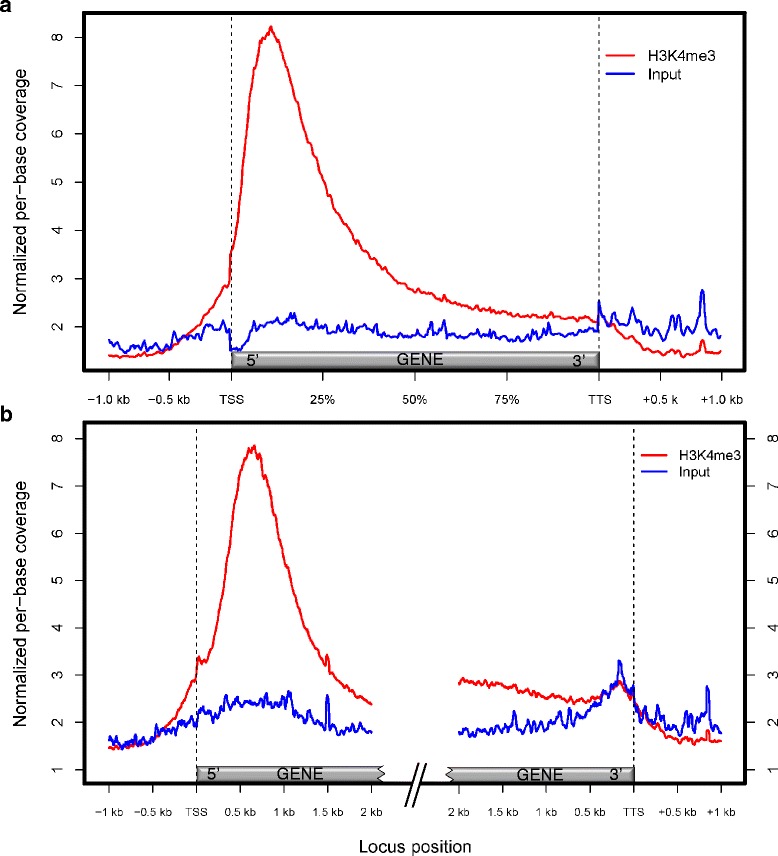
H3K4me3 and Input ChIP-seq profiles across the 1 kb promoter, transcribed and 1 kb downstream regions of annotated loci. **(a)** Bin-wise, showing relative gene length. **(b)** Absolute distance anchored at the 5’ and 3’ ends of transcribed regions. The 5’ and 3’ regions were analysed separately, thus profiles overlap for genes < 4 kb. Per-base coverage values were normalized between H3K4me3 and Input libraries. TSS, transcription start site; TTS, transcription termination site; gene, regions annotated as transcribed in *E. grandis* v.1.1.

We reconstructed the binding profile of H3K4me3 relative to genic regions by calculating per-base coverage of H3K4me3 and input libraries across all annotated genes, as well as the upstream and downstream sequences, in a bin-wise manner. As expected, H3K4me3-enriched library coverage peaked shortly after the TSS (Figure 2a). In contrast, input coverage was comparatively uniform across transcribed regions and their flanking non-coding sequences (Figure 2a). Similarly, when absolute distance relative to the TSS or TTS (transcription termination site) was analysed for H3K4me3 and input coverage across genes, the H3K4me3 profile yielded a prominent peak ~600-700 bp downstream of the TSS (Figure 2b). The position of the peak was similar for genes of different lengths (Additional file 2: Figure S13).

### Expression dynamics of H3K4me3-enriched genes

**Figure 3:**
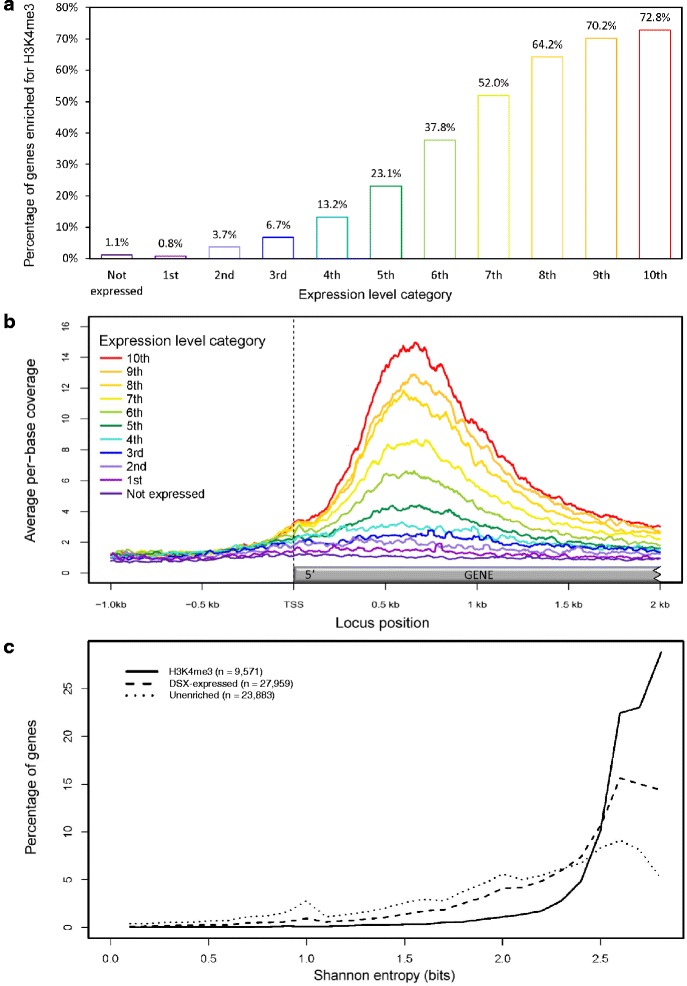
Expression properties associated with H3K4me3 enrichment in developing secondary xylem tissue. **(a)** Percentage of genes enriched for H3K4me3 among non-expressed genes and genes with increasing expression levels, represented as ten ordinal categories of similar size (n ≈ 2,760). **(b)** H3K4me3 enrichment (library coverage) at the 5’ regions of transcribed genes, for each of the expression level categories in **(a)**. Average per-base coverage values from 1 kb upstream to 2 kb downstream of the transcriptional start site (TSS) is shown for each expression level category. **(c)** Tissue specificity of genes enriched for H3K4me3 (solid line), genes expressed in developing secondary xylem regardless of histone modification status (dashed), and genes expressed in developing secondary xylem but lacking H3K4me3 modification (dotted), as measured by Shannon entropy. High entropy values indicate broad, even expression across tissues; low values indicate high tissue specificity. The maximum possible entropy value for this data is 2.81.

H3K4me3 enrichment of genes is tightly associated with their corresponding transcript abundances [55]. We investigated the relationship between H3K4me3 modification of genes and their RNA-seq expression values in DSX tissue collected from a different trial [56]. The sample collection, data analysis and results of this experiment are discussed in Vining *et al.* [57]*.* On average, genes enriched for H3K4me3 were expressed almost two-fold higher than the full set of annotated genes with detected expression in DSX, and over five-fold more than those lacking the histone modification (Additional file 2: Figure S14). Less than one percent of H3K4me3-enriched genes had no expression evidence (not shown). After ranking expressed genes by transcript abundance and dividing them into ten ordinal expression level categories of equal size (~2760 genes per category), the percentage of genes exhibiting H3K4 trimethylation increased with gene expression levels (Figure 3a). Of the top tenth of genes expressed in DSX, 72.8% were trimethylated at H3K4, compared to 1.1% of genes with no detected expression (Figure 3a). These results indicate that H3K4me3 enrichment of genes is indeed predictive of gene activation, where H3K4me3 is most often associated with genes expressed at high levels.

We next investigated whether local coverage of mapped H3K4me3 ChIP-seq reads, which reflects the degree of enrichment of H3K4 trimethylation at a given locus, is related to transcript levels. Average H3K4me3 ChIP-seq library coverage was calculated for each base around the 5’ regions of genes for each expression level category in Figure 3a. As expected, we found that H3K4me3 enrichment was most pronounced around the 5’ region of genes in the top expression level category, showing a concordant decrease with less abundant transcript levels (Figure 3b). This relationship was maintained throughout the 2 kb region downstream of the TSS (Figure 3b). These results confirm that the degree of H3K4 trimethylation at a locus is correlated with transcript abundance in *Eucalyptus* DSX.

In addition to an association with gene expression, it was reported in *Arabidopsis thaliana* that genes enriched for H3K4me3 tended to be less tissue-specific than those lacking the H3K4me3 modification, regardless of H3K4 mono- or dimethylation states [27]. To further explore the relationship between H3K4me3 modification and expression in *Eucalyptus*, Shannon entropy values [58,59] of relative transcript abundance across seven tissues and organs [56] were calculated for the 9,571 genes that were expressed in at least one tissue and overlapped a significant H3K4me3 peak, and compared to entropy values for (1) all genes expressed in DSX, and (2) expressed genes that were not significantly enriched for H3K4me3. Genes enriched for H3K4me3 had significantly higher entropy values (i.e., lower tissue specificity) compared to both the expressed, and expressed but lacking H3K4me3, gene sets (Kolmogorov-Smirnov test, *P* < 2.2 × 10^−16^) (Figure 3c). Similarly, genes lacking the H3K4me3 mark were significantly more tissue-specific than all expressed genes in DSX (*P* < 2.2 × 10^−16^; Figure 3c). Thus, H3K4me3-enriched genes tend not only to be highly expressed, but also show less tissue specificity in general than unenriched genes in *Eucalyptus*. It is noteworthy, however, that a large proportion (34%) of H3K4me3-enriched genes had entropy values lower than the average of 2.54 for genes expressed in DSX (i.e. high tissue/organ-specificity). Furthermore, the average relative expression of genes in DSX (that is, the proportion of total transcript detected in DSX compared to all seven tissues) was higher for H3K4me3-enriched genes (17.9%) than unenriched genes (11.7%) and the total DSX transcriptome (13.5%; expected value is 14.3%). This tissue bias is consistent with the association of H3K4me3 with transcriptional activation in the sampled tissue, and suggests that H3K4 trimethylation occurs at genes with strong, broad expression, as well as with expressed genes preferentially expressed in DSX.

### The role of H3K4me3 modification in regulating wood-related biological processes

Since the 9,623 genes enriched for H3K4me3 in DSX comprise over 26% of those in the v.1.1. annotation and tend to be more broadly expressed than those lacking the modification, it was hypothesized that H3K4me3-enriched genes would be overrepresented for general biological processes rather than those specific to wood formation. Since H3K4me3 is strongly associated with transcribed genes, we used as the reference set all genes transcribed in DSX tissue to assess whether those enriched for H3K4me3 genes showed over- or underrepresentation of particular biological functions represented in this set. As expected, broad biological functions such as translation, protein metabolism and catabolism, primary metabolism and mRNA metabolism were significantly overrepresented among H3K4-trimethylated genes (Additional file 3: Table S4). Interestingly, relative to genes expressed in DSX tissue, phenylpropanoid biosynthesis, responses to biotic and abiotic stress and a number of regulatory processes were significantly underrepresented among H3K4me3-trimethylated genes (Additional file 3: Table S4).

**Figure 4:**
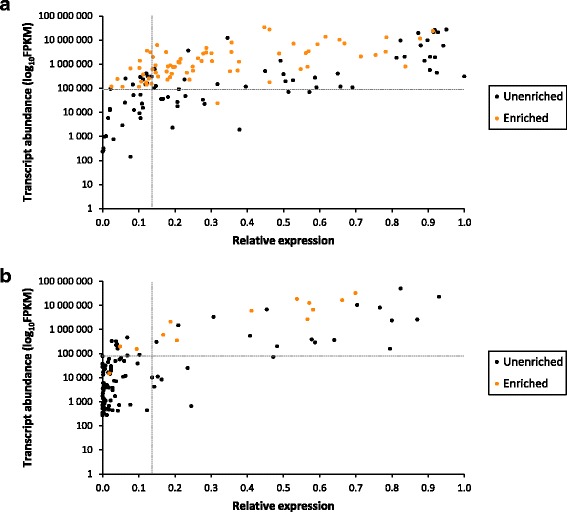
Association of H3K4me3 secondary cell wall candidate genes in *E. grandis*. **(a)** Cellulose and xylan biosynthesis. **(b)** Phenylpropanoid biosynthesis **(b)**. Genes enriched (orange dots) or unenriched (black dots) for H3K4me3 were plotted by absolute transcript abundance in DSX tissue (*y-*axis; median FPKM value of 89,300 indicated) and relative transcript abundance in DSX tissue compared to shoot tips, young leaves, mature leaves, flowers, roots and phloem (*x-*axis; expected value of 0.142 indicated). The full gene lists are presented in Additional file 3: Table S5, Table S6.

While GO terms characteristic of xylogenesis, such as secondary cell wall biosynthetic processes, were not overrepresented among H3K4me3-enriched genes, H3K4 trimethylation at genes involved in xylogenesis provides insights into how they are regulated at the chromatin level. A substantial proportion (~43%) of annotated functional homologs of cellulose and xylan biosynthesis-associated genes [51] were enriched for H3K4me3 (Additional file 3: Table S5), most of which were highly and preferentially expressed in DSX tissue (Figure 4a). A smaller proportion (~8%) of phenylpropanoid pathway genes overlapped H3K4me3 peaks owing to a large number of tandemly duplicated homologs with low transcript abundance (Additional file 3: Table S6), possibly explaining the significant underrepresentation of this pathway among H3K4me3-enriched genes. Only considering phenylpropanoid pathway genes expressed above the median FPKM level, ~55% were enriched for H3K4me3 (Figure 4b). Mapping of nearest *Arabidopsis thaliana* homologs of H3K4me3-enriched *Eucalyptus* genes and their corresponding transcript abundance in DSX to the KEGG phenylpropanoid metabolism pathway ath00940; [60] showed that most of the central monolignol biosynthetic enzymes were H3K4-trimethylated (Additional file 2: Figure S15). This suggests a biologically relevant role for H3K4me3 in the regulation of the phenylpropanoid pathway.

**Figure 5:**
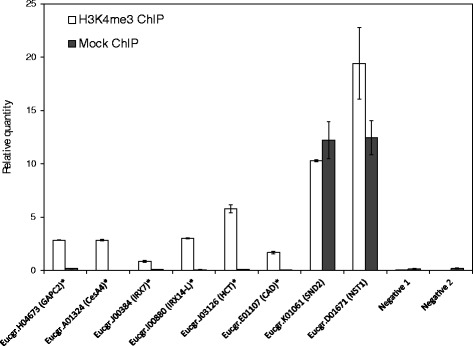
ChIP-qPCR validation of H3K4me3-enriched and control loci. The putative *Arabidopsis* ortholog of each candidate is indicated in parenthesis. Asterisks denote H3K4me3 targets identified in the ChIP-seq analysis. Eucgr.K01061 and Eucgr.D01671 serve as validations of identified false positives arising from nonspecific binding. Two intergenic negative control regions are included. Error bars indicate standard deviation of three technical replicates.

To validate the ChIP-seq data, we performed a ChIP-qPCR analysis focusing on carbohydrate and secondary cell wall-associated loci with evidence of H3K4 trimethylation. This method evaluates enrichment directly against mock (nonspecific IgG) ChIP, whereas the ChIP-seq peak-calling algorithm uses input as negative control, thus providing an independent assessment of enrichment. All six positive regions identified by ChIP-seq and assayed by ChIP-qPCR showed clear immunoprecipitation enrichment (9–165 fold) in the H3K4me3 ChIP sample compared to mock ChIP (Figure 5; asterisks). We included two controls for the qPCR analysis. In the first, we validated two false positive H3K4me3 regions overlapping homologs of *SND2* and *NST1* identified through MACS analysis of the IgG_2a_ (mock ChIP) library. These targets showed similar amplification between H3K4me3 and mock ChIP samples as expected (Figure 5). Second, we profiled two intergenic negative control regions which showed negligible amplification in both H3K4me3 and mock ChIP samples, showing that there was no template loading bias in the H3K4me3 samples (Figure 5).

## Discussion

In this study, we sought to explore the role of H3K4me3 in the epigenomic regulation of secondary xylem development in *E. grandis*, modifying and optimizing existing chromatin preparation protocols in order to perform ChIP-seq on this challenging tissue. Over 80% of identified peaks were shared between sampled individuals at a stringent IDR (Additional file 3: Table S2), showing that our approach successfully captured biologically relevant binding events. We have shown that high-quality ChIP-seq profiles of developing xylem collected from mature field-grown trees can be generated using our approach, revealing both known properties of trimethylated H3K4 as well as a novel role in the epigenomic regulation of various aspects of xylogenesis.

While the use of a ChIP DNA amplification step allowed for the preparation of Illumina sequencing libraries from only 1–2 ng, or less, of ChIP DNA in this study, the relatively high proportion of redundant sequences arising from template amplification (Additional file 3: Table S1) is undesirable. We have also frequently found that most of the DNA in amplified samples was >500 bp in length (Additional file 2: Figure S7), resulting in libraries with a small fraction of the DNA having the preferred insert size of 100–500 bp. These limitations favour the preparation of Illumina libraries from unamplified ChIP DNA, where pooling of technical replicates may be necessary to obtain enough ChIP DNA for successful library construction.

In *Eucalyptus*, H3K4 trimethylation generally occurs ~600 - 700 bp downstream of annotated TSSs (Figure 2b), irrespective of the gene length, but we point out that this value is dependent on the accuracy of TSS predictions in the *E. grandis* v.1.1. genome annotation. Nonetheless, the observed range is similar to that reported in rice [30], while further from the TSS than that in *Arabidopsis*, which mostly occurs within 500 bp of the TSS [27,28]. The vast majority of H3K4me3 peaks were gene-associated (Figure 1), including noncoding RNA genes that are predicted to be transcribed by Pol II (Table 1), and the H3K4me3 ChIP-seq library coverage within the first kilobase after the TSS correlated well with transcript abundance (Figure 3b). As transcript levels increased, a greater proportion of genes expressed at each level became enriched for H3K4me3 (Figure 3a), supporting the known function of H3K4me3 in keeping expressed genes in a transcriptionally active state [11,23]. The H3K4me3 signal at a given locus could represent the degree of H3K4me3 trimethylation in one particular cell type, and/or the proportion of cell types in the tissue that are H3K4-trimethylated at that locus. ChIP-seq analysis of individual xylem cell types remains a future challenge.

H3K4me3 peaks predicted on-off states of target genes to a high degree of precision: over 99% of H3K4me3-enriched genes were expressed in DSX tissue, >85% of them above the median FPKM value, and only ~1% of genes without evidence of expression were positive for H3K4me3. Considering that our RNA-seq data originated from an independent trial, the exceptions to the rule are unsurprising. Conversely, gene transcript level was not necessarily predictive of H3K4me3 modification at a locus – even among the most highly expressed genes in DSX tissue, ~27% did not show evidence of H3K4me3 enrichment (Figure 3a). While increased ChIP-seq sequencing depth may detect more H3K4me3 binding events, accurate prediction of mRNA abundance generally requires information for more than one histone modification mark [61] and depends largely on transcript quantification methods (e.g. CAGE, RNA-Seq) [62]. It is likely that partially functionally redundant histone modifications, such as mono- or dimethylated H3K4 or lysine 9-acetylated histone H3, may be sufficient to promote an active chromatin configuration in the absence of H3K4me3.

It was reported in *Arabidopsis thaliana* that H3K4me3-modified genes tend to show less tissue-specificity compared to genes lacking the mark [27,28], a trend we confirmed in *Eucalyptus* (Figure 3c). In light of this, the overrepresentation of general cellular processes and “housekeeping” functions among H3K4me3-associated genes relative to DSX-expressed transcripts (Additional file 3: Table S4) is expected. Despite this tendency, we showed that H3K4me3 was present at several highly expressed genes involved in secondary cell wall biosynthesis which were also preferentially expressed in DSX (Additional file 3: Table S5, Table S6). Thus, H3K4 trimethylation appears to play a role in the epigenomic regulation of wood formation. It is likely that H3K4me3 modification is employed to keep highly expressed genes in an active state once activated in a given tissue or cell type, in this case DSX. The lower tissue specificity of H3K4me3-enriched genes is probably a reflection of a general negative correlation between tissue-specificity and gene expression level [63,64]. For example, the top 10% of genes expressed in DSX in the RNA-seq dataset used in this study had significantly higher average entropy than the entire DSX transcriptome (not shown).

H3K4 trimethylation profiles, especially when combined with DNase-seq data [26], are a useful resource for annotating TSSs as well as direction of transcription [65]. Our H3K4me3 data suggest that 196 low-confidence gene models in the v.1.0 annotation that were removed in the v.1.1 annotation are potentially true gene models. We suggest that these gene models could be prioritized based on both RNA-seq coverage as well as H3K4me3 fold enrichment provided in Additional file 6. We have found numerous examples of H3K4me3 peaks located at genomic regions that have not been previously annotated, but show clear RNA-seq expression coverage (see Additional file 2: Figure S16 for three examples). Thus, the H3K4me3 data from this study is an important line of evidence for future revisions of the *E. grandis* genome annotation.

## Conclusions

ChIP-seq has proved to be a valuable technique for the high-throughput analysis of *in vivo* protein-DNA interactions in yeast, mammals and, to an increasing extent, plants. As this technology becomes more widespread, its application to novel and challenging tissues will require additional optimization and testing. ChIP-seq combined with a nano-ChIP-seq protocol allowed us to produce high-quality profiles of a modified histone in developing secondary xylem tissue, here in mature *Eucalyptus* trees, closely following standards recommended by the ENCODE Consortium [48]. The 12,177 H3K4me3 peaks identified in this study mostly overlapped the 5’ vicinity of transcribed regions, the enrichment of which was strongly correlated with gene expression. While H3K4me3-enriched genes tend to be broadly expressed across tissues, this epigenomic mark is associated with highly expressed, tissue-specific genes with crucial functions in wood formation. The H3K4me3-enriched miRNAs and snoRNAs identified in this study suggest that these noncoding RNAs are biologically active in developing secondary xylem, guiding future research into the post-transcriptional regulation of wood formation. Finally, a number of H3K4me3 peaks were located at unannotated genomic regions with transcriptional evidence, providing a valuable resource for improved annotation of the *E. grandis* genome sequence. Epigenomic profiles such as modified histone distributions have important implications for how we understand and interpret genome function. This study probes the poorly understood role of chromatin organization during xylogenesis and promotes further investigation into the functions of epigenomic features in plants. Researchers can visualize H3K4me3 ChIP-seq data reported here in a custom *E. grandis* genome browser in EucGenIE [56].

## Methods

### Plant materials

ChIP-seq experiments were performed on *E. grandis* clone TAG0014 (Mondi Tree Improvement Research, KwaMbonambi, South Africa). DSX scrapings from seven-year-old ramets growing in clonal trial in KwaMbonambi, KwaZulu-Natal Province, South Africa were sampled in September 2012 (early spring). The bark was peeled off at breast height to expose the DSX tissue of two individuals, V5 and V11. 1–2 mm was lightly and uniformly scraped off using a razor, gently squeezed of excess sap and immediately flash-frozen in liquid nitrogen. Samples were stored at −80°C until use.

### Chromatin fixation, isolation and sonication

Nuclei were purified as described by Kaufmann *et al.* [44], with modifications. Frozen DSX tissue was ground using a model A 11 basic analytical mill (IKA, Germany) followed by fine grinding in liquid nitrogen using a mortar and pestle. Every five grams of frozen, ground DSX tissue was fixed in 25 ml M1 buffer supplemented with 1% formaldehyde, 1 mM EDTA and 1 mM phenylmethanesulfonyl fluoride (PMSF) on ice for 30 min. Fixation was quenched with 1/10 volume 1.25 M glycine for 5 min on ice, followed by addition of M1 buffer without formaldehyde to 50 ml. The suspension was filtered through 60 μm nylon mesh wetted with M1 buffer, changing the filter at least once per 50 ml suspension, and again through a double 60 μm nylon mesh. After centrifugation at 1,000 × *g* for 20 min (4°C), the pellet was resuspended in 25 ml ice-cold M2 buffer containing 1 mM PMSF and Complete Protease Inhibitor cocktail (CPIC; Roche), centrifuged at 1,000 × *g* for 10 min at 4°C and resuspended in 25 ml ice-cold M3 buffer supplemented with 1 mM PMSF and CPIC. After centrifugation similarly for 10 min, the nuclear pellet was resuspended in ~1.5 ml sonic buffer containing 1 mM PMSF and CPIC. Sonication was performed on 250 μl crude chromatin per 1.5 ml tube on ice using a Branson Sonifier 450 probe sonicator with 20 pulses of 10 s duration on setting 1, and >30 s rest on ice between pulses. Samples were mixed every ten cycles. After sonication, samples were centrifuged twice at 16,000 × *g* (10 min, 4°C) and stored at −80°C.

### Micrococcal nuclease (S7) assay

Frozen DSX tissue (2 g) was ground to fine powder in liquid nitrogen. Nuclei were isolated as described above, excluding formaldehyde crosslinking and the addition of sonic buffer. The crude nuclear pellet was resuspended in 350 μl nuclei digestion buffer [66] containing 400 μg RNase A. Samples were divided equally into four tubes and incubated with 0, 5, 10 or 20 U of Nuclease S7 (Roche) at 37°C for 15 min. Hydrolysis was terminated with 5 mM EDTA. Nuclei were lysed with 0.5% SDS and centrifuged (20,000 × *g*, 5 min) to clear. Soluble DNA was purified using the Nucleospin PCR purification kit (Macherey-Nagel, Düren, Germany).

### Protein extraction and Western blot analysis

Nuclei were purified according to the method of Kaufmann *et al.* [44], with modifications. DSX was ground in liquid nitrogen and suspended in M1 buffer containing 1 mM PMSF and 1 mM EDTA at 5 ml per gram of tissue, for 30 min. The suspension was filtered twice through 60 μm nylon mesh and pelleted at 1000 × *g* (20 min, 4°C). The pellet was resuspended in 5 ml M2 buffer supplemented with 1 mM PSMF and CPIC, re-pelleted (1000 × *g*, 10 min, 4°C) and resuspended in 250 ul M3 buffer containing 1.7 M sucrose and CPIC. The suspension was overlaid on 1.5 ml 1.7 M sucrose in M3 buffer and centrifuged for 40 min at 16,000 × *g* (4°C). The pellet was resuspended in 1 ml M3 to wash, re-pelleted (12,000 × *g*, 5 min, 4°C) and the remaining pellet resuspended in 1 pellet volume of extraction buffer (10 mM sodium phosphate buffer pH 7.0, 150 mM NaCl, 0.1 mM EDTA, 5% glycerol, 10 mM β-mercaptoethanol, 0.1 mM PMSF, CPIC). The pellet was briefly sonicated with a Branson 450 sonicator (30s, 10% power output) and gently vortexed for 30 min at 4°C. Soluble protein in the supernatant from two rounds of centrifugation (16 000 × *g*, 10 min, 4°C) was quantified using the Qubit Protein Assay Kit (Invitrogen), subjected to denaturing electrophoresis on a 12% SDS-PAGE gel and transferred to a nitrocellulose membrane using the semidry method. Blots were blocked with 5% nonfat milk, probed with 1:2000 dilution of anti-H3K4me3 antibody (Millipore #07-473) overnight (4°C) and incubated with horseradish peroxidase-conjugated goat anti-rabbit secondary antibody (Cappel Laboratories Inc., PA). Blots were treated with SuperSignal West Pico Chemiluminescent substrate (Thermo Scientific, Rockford, IL) and developed with CL-XPosure film (Thermo Scientific).

### Chromatin immunoprecipitation, DNA amplification and sequencing

A minimum of 3 μg *E. grandis* DSX chromatin was incubated with 1 μg anti-H3K4me3 antibody (Millipore #07-473), or 1 μg naïve mouse IgG_2a_ (sc-3878, Santa Cruz Biotechnology, CA) as negative control, overnight at 4°C. Chromatin immunoprecipitation was performed as described by Adli & Bernstein [45] using 40 μl protein A-agarose beads, 25% slurry (sc-2001, Santa Cruz Biotechnology, CA). After crosslink reversal and DNA purification, the ChIP DNA was quantified with the Qubit HS dsDNA kit (Invitrogen). A minimum of 1 ng ChIP or input DNA was amplified according to the protocol of Adli & Bernstein [45], with modifications. We replaced the use of Sequenase v.2.0 DNA polymerase (Affymetrix, CA) with *Bsu* DNA polymerase, large fragment (NEB, MA), and substituted the corresponding Sequenase reaction buffer with NEB Buffer 2. We used 2 U of *Bsu* DNA polymerase per pre-amplification cycle, extended the pre-amplification extension time to 20 min and used 32 pmol P1 primer. Both the pre-amplification and PCR reactions were supplemented with 50 ng/μl tRNA. We applied a generic ExoSAP cocktail by adding 0.5 U rAPID alkaline phosphatase (Roche Applied Science, Ltd) and 5 U *E. coli* Exonuclease I (NEB), incubating at 37°C for 30 min and heat-inactivating the enzymes at 80°C for 20 min. For the Phusion PCR reactions we used 4 ul 10 mM dNTPs and 0.5 ul Phusion DNA polymerase per 50 ul reaction. PCR extension time was reduced to 5 s. Amplified DNA was digested with BciVI to yield 3’ adenosine overhangs; 20 ng template was ligated to Illumina primers for library preparation and DNA sequencing (Beijing Genome Institute, Hong Kong), generating 50 nt paired-end sequences.

### Bioinformatics analysis

Sequence data were trimmed of primer and adapter sequences and purged of low-quality reads (phred score <20 for ≥50% of the read, or reads with >10% “N” bases). In some cases further trimming of the 5’ end was required to reduce overrepresented k-mers as identified using FastQC [67]. The reads were mapped to the *E. grandis* v.1.1 reference genome [47] using Bowtie2 [68] in Galaxy [69,70], using parameters: “sensitive” pre-set option, 50–1,000 bp insert size for a valid PE pair, end-to-end alignment. BAM alignments of ChIP-seq and input libraries were converted to BED format and subjected to peak-calling analysis using Model-based Analysis of ChIP-Seq (MACS) v.2 [50], with input libraries as controls and where paired-end reads were treated as single reads, duplicate reads were discarded, effective genome size was 640 Mb, initial *P-*value set at 0.01, band width set to 300 bp and peak-calling model based on 5 - 15-fold enrichment. Input library sequence depths were allowed to exceed those of the ChIP libraries in order to increase peak-calling specificity [71]. To assess replicate consistency, the Irreproducible Discovery Rate (IDR) analysis [49,72] was performed on biological replicates (IDR < 0.02), pseudoreplicates of each sample (IDR < 0.02) and pseudoreplicates of pooled biological replicates (IDR < 0.01), each ranked by *P*-value, using the idrCode R package (//sites.google.com/site/anshulkundaje/projects/idr#TOC-IDR-PIPELINE). Significant H3K4me3 peaks were identified from the pooled samples using IDR < 0.01, and further constrained based on IDR < 0.05 between biological replicates. To exclude false positive signals, 261 H3K4me3 peaks that overlapped the 2,362 peaks called in the IgG_2a_ ChIP-seq negative control by the same criteria were removed from all analyses. H3K4me3-enriched genes were defined as those overlapping a significant H3K4me3 peak by at least one base. Strand cross-correlation analysis was performed using SPP [73]. Shannon entropy [58] was calculated for each gene as described by Schug *et al*. [59], using previously obtained RNA-seq data [56]. All RNA-seq data are available at http://eucgenie.bi.up.ac.za. Genes were considered expressed if they had an FPKM value above 70. Coverage distributions across genomic coordinates were calculated using the BEDTools suite [74] based on bulked BAM files of both individuals. Using the TSS and TTS of genes as anchors, 1 kb promoter or downstream regions plus 2 kb into each gene model was delineated using slopBed. Truncated regions (<3 kb) were discarded. Per-base coverage was calculated for each locus using coverageBed, pooled across all loci for each position relative to the TSS or TTS and normalized based on sequencing depth. For the peak and genomic feature overlap analysis, CDS, 5’ UTR, 3’ UTR and gene annotations in the v.1.1 annotation were made nonredundant using mergeBed, and introns delineated by subtracting the nonredundant CDS annotations from the “gene” set using subtractBed. The overlap (bp) between the total set of H3K4me3 peak intervals and each genomic feature was calculated separately using intersectBed. For Gene Ontology analysis, the nearest *Arabidopsis* BLASTP hits of H3K4me3-enriched or DSX-expressed genes were analysed for Biological Process enrichment analysis (Bonferroni-adjusted *P*-value < 0.05) using GOToolBox [75]. miRNA putative targets were identified using psRNATarget [76].

### Quantitative polymerase chain reaction (qPCR)

The V11 ChIP-seq samples (prior to library preparation) were used for ChIP-qPCR analysis. Primers targeting selected genomic regions are listed in Additional file 3: Table S7. Sample concentrations were quantified with the Qubit HS dsDNA kit (Invitrogen) and equal quantities of input, H3K4me3 ChIP, and mock ChIP (i.e. IgG_2a_) DNA added to triplicate technical repeat reactions for qPCR quantification and melting curve analysis using the LightCycler 480 [50 cycles of 95°C denaturation (10s), 60°C annealing (10s) and 72°C extension (15s)] (Roche, Switzerland). Crossing points (Cp) were calculated using the second derivative maximum method and quantities relative to the input sample calculated using the formula E^ΔCp(input) - Cp(sample)^, where E is the efficiency calculated from a standard curve of the relevant primer set.

### Availability of supporting data

The datasets supporting the results of this article are available in the NCBI’s Gene Expression Omnibus [77], Series accession number GSE67873 (http://www.ncbi.nlm.nih.gov/geo/query/acc.cgi?acc=GSE67873).

## Additional files

Additional file 1:
**Supplementary Note S1.**
Additional file 2:
**Figure S1, Figure S2, Figure S3, Figure S4, Figure S5, Figure S6, Figure S6, Figure S7, Figure S8, Figure S9, Figure S10, Figure S11, Figure S12, Figure S13, Figure S14, Figure S15, Figure S16.**
Additional file 3:
**Table S1, Table S2, Table S3, Table S4, Table S5, Table S6, Table S7.**
Additional file 4:
**Genomic locations and fragment coverage of significant H3K4me3 peaks.**
Additional file 5:
**Genomic locations of annotated genes overlapping with significant H3K4me3 peaks.**
Additional file 6:
**Genomic locations of low-confidence gene models overlapping with significant H3K4me3 peaks.**

## Abbreviations

CPIC: Complete protease inhibitor cocktail
DSX: Developing secondary xylem
GO: Gene ontology
H3K4me3: Lysine 4-trimethylated histone H3
miRNA: microRNA
ncRNA: noncoding RNA
Pol II: RNA polymerase II
snRNA: small nuclear RNA
snoRNA: small nucleolar RNA
sRNA: small RNA
TTS: Transcription termination site
TSS: Transcription start site
