# Supplementary material for: Genome-wide mapping of histone H3 lysine 4 trimethylation in Eucalyptus grandis developing xylem
Source: BMC Plant Biol. 2015 May 10;15:117. doi: 10.1186/s12870-015-0499-0 (PMC4425858; doi:10.1186/s12870-015-0499-0)
Supplement: Additional file 1: — Supplementary Note S1. [file 12870_2015_499_MOESM1_ESM.pdf]

Supplementary Note S1

Optimisation of chromatin isolation from *E. grandis* developing xylem

We used the protocol by Kaufmann *et al*. [1] for nuclei isolations, with modifications as described in Methods. Our decision was based on a high yield of nuclear DNA obtained using this protocol compared to those of McKeown *et al.* [2], Saleh *et al.* [3] and Loureiro *et al.* [4] (Additional file 2, Figure S1). We investigated whether intact chromatin could be isolated from *E. grandis* DSX using this method as assessed by the persistence of histone-DNA associations. Micrococcal nuclease (MNase) was used to detect nucleosomes by virtue of its ability to cleave the linker region between nucleosomes, but rendering DNA packaged within nucleosomes intact. Whereas naked genomic DNA was completely degraded in the presence of 10 U MNase, DSX chromatin exposed to up to 20 U MNase was hydrolysed into distinct nucleosomal fragments (Additional file 2, Figure S2). This pattern is consistent with successive cleavage of linker DNA between nucleosomes, liberating nucleosomal fragments in decreasing dividends of ~195 bp to ~140 bp [5]. These results indicate that intact chromatin was successfully isolated from DSX tissue.

Formaldehyde-crosslinked chromatin is generally fragmented using sonication prior to ChIP [6]. We optimized sonication parameters for DSX chromatin to produce an average fragment size suitable for ChIP-seq library preparation (~200 - 500 bp). Favouring a low sonication output and large number of pulses, we found that twenty pulses of 10s with a probe sonicator at 10% power output produced the desired fragment range (Additional file 2, Figure S3a). Analysis of DNA in the residual nuclear pellet showed that this sonication treatment released most of the chromatin (Additional file 2, Figure S3b).

The degree of formaldehyde crosslinking is critical for ChIP-seq since insufficient crosslinking may result in loss of bound proteins and crosslink reversal during sonication, while excessive crosslinking compromises protein-antibody recognition and reduces DNA yield after crosslink reversal [7]. To optimize crosslinking conditions, we fixed frozen and ground DSX tissue from field-grown *E. grandis* trees for varying durations in 1% formaldehyde buffer chilled on ice to prevent protein degradation, and purified nuclei. We sonicated chilled samples to assess the degree of crosslink reversal as a by-product of sonication. We found that 30 min fixation gave the best trade-off between durable crosslinking and DNA yield following crosslink reversal (Additional file 2, Figure S4).

References

1. Kaufmann K, Muiño J, Østerås M, Farinelli L, Krajewski P, Angenent GC: **Chromatin immunoprecipitation (ChIP) of plant transcription factors followed by sequencing (ChIP-SEQ) or hybridization to whole genome arrays (ChIP-CHIP)**. *Nature Protocols* 2010, **5**:457–472.
2. McKeown P, Pendle AF, Shaw PJ: **Preparation of *Arabidopsis* nuclei and nucleoli**. In: *The Nucleus: Nuclei and Subnuclear Components.* Humana Press; 2008: 65-74.
3. Saleh A, Alvarez-Venegas R, Avramova Z: **An efficient chromatin immunoprecipitation (ChIP) protocol for studying histone modifications in *Arabidopsis* plants**. *Nature Protocols* 2008, **3**(6):1018-1025.
4. Loureiro J, Rodriguez E, Doležel J, Santos C: **Two new nuclear isolation buffers for plant DNA flow cytometry: A test with 37 species**. *Annals of Botany* 2007, **100**:875–888.
5. Philipps G, Gigot C: **DNA associated with nucleosomes in plants**. *Nucleic Acids Research* 1977, **4**(10):3617-3626.
6. Das PM, Ramachandran K, vanWert J, Singal R: **Chromatin immunoprecipitation assay**. *Biotechniques* 2004, **37**:961-969.
7. Haring M, Offermann S, Danker T, Horst I, Peterhansel C, Stam M: **Chromatin immunoprecipitation: optimization, quantitative analysis and data normalization**. *Plant Methods* 2007, **3**:11.
