# Supplementary material for: Genome-wide mapping of histone H3 lysine 4 trimethylation in Eucalyptus grandis developing xylem
Source: BMC Plant Biol. 2015 May 10;15:117. doi: 10.1186/s12870-015-0499-0 (PMC4425858; doi:10.1186/s12870-015-0499-0)
Supplement: Additional file 2: — Figure S1, Figure S2, Figure S3, Figure S4, Figure S5, Figure S6, Figure S6, Figure S7, Figure S8, Figure S9, Figure S10, Figure S11, Figure S12, Figure S13, Figure S14, Figure S15, Figure S16. [file 12870_2015_499_MOESM2_ESM.pdf]

Supplementary figures

a

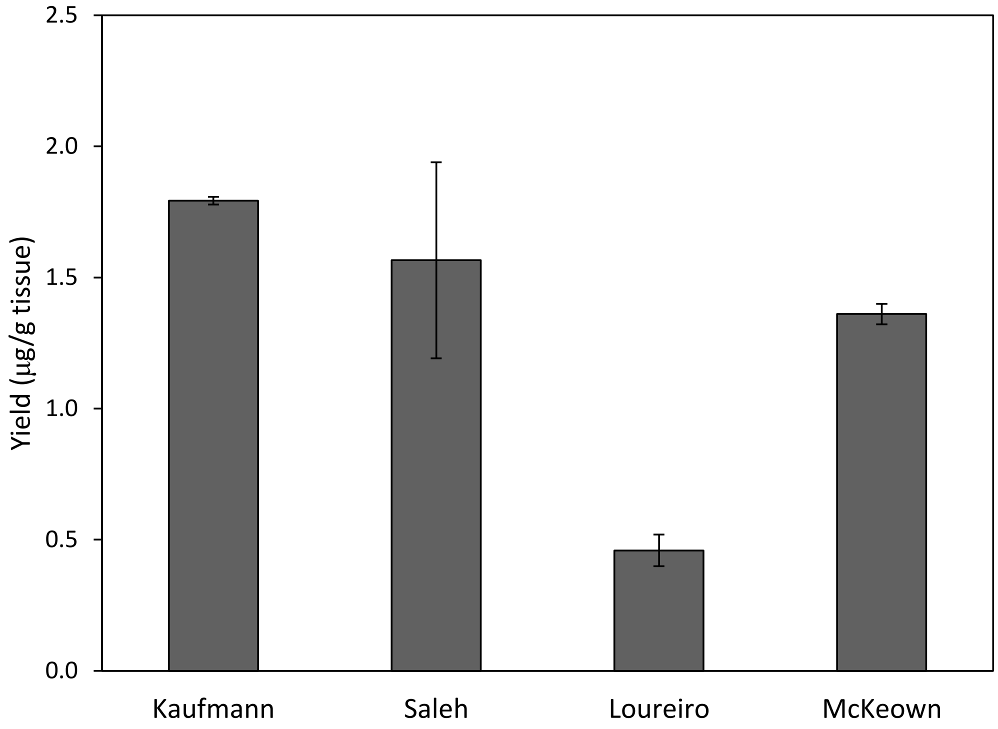

b

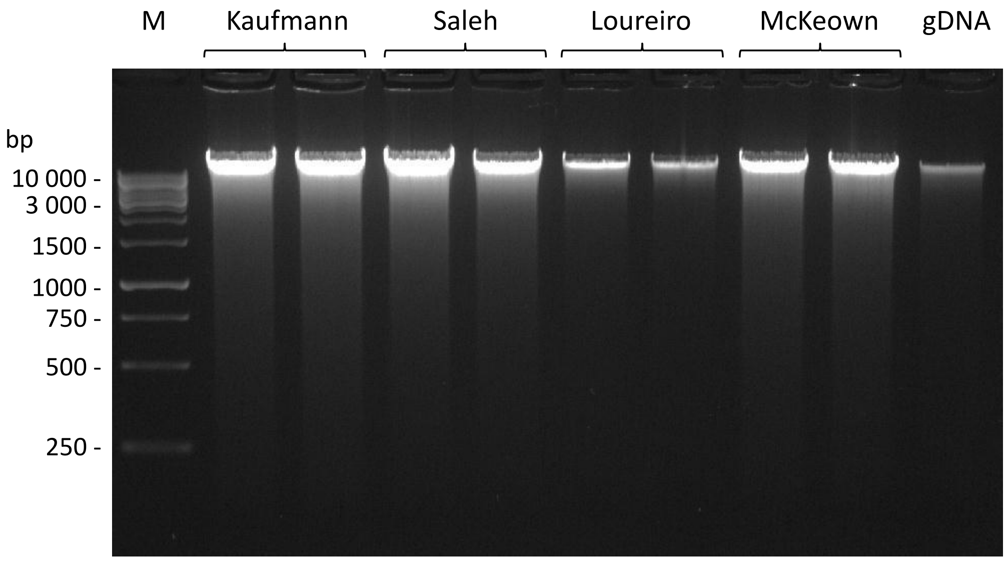

Figure S1. Assessment of DNA yield from crude nuclei preparations of *E. grandis* developing secondary xylem tissue using different buffers [1-4]. (a) Nuclear DNA yield from crude nuclear pellets. Error bars indicate the range of two extractions. **(b)** Agarose gel electrophoresis analysis of nuclear DNA quality. Duplicate extractions were performed for each buffer. M, GeneRuler 1 kb DNA ladder (Fermentas). gDNA, genomic DNA extraction from whole developing secondary xylem tissue.

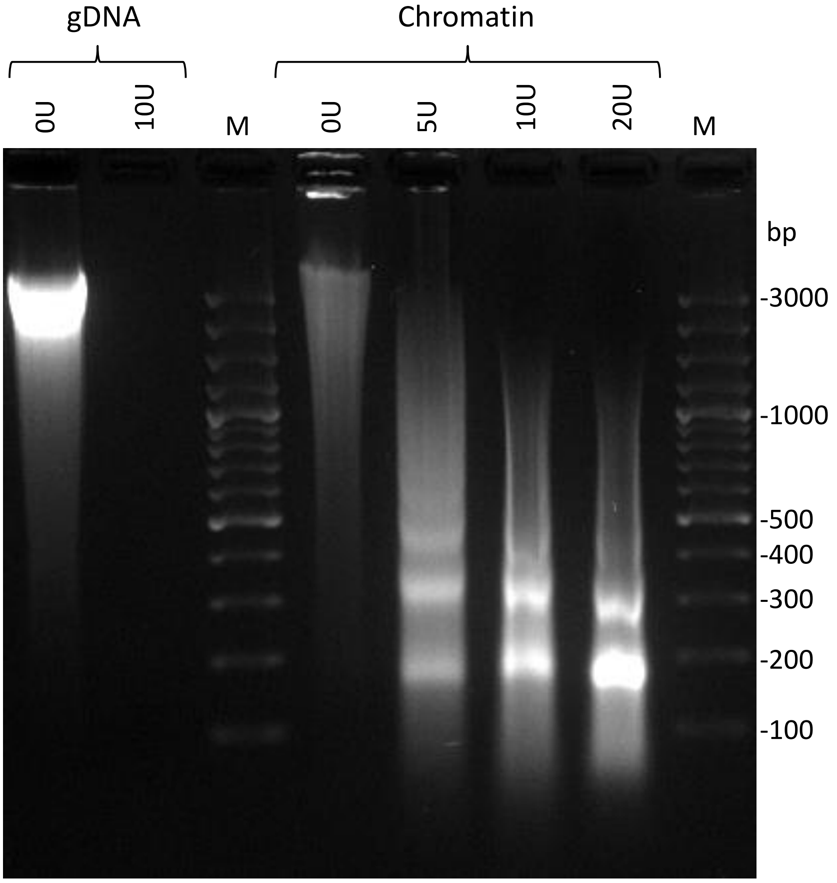

Figure S2. Agarose gel electrophoresis analysis of micrococcal nuclease-digested chromatin isolated from developing secondary xylem. Genomic DNA (gDNA) and chromatin from developing secondary xylem were exposed to increasing units (U) of micrococcal nuclease. M, GeneRuler 100 bp plus DNA ladder (0.5 µg).

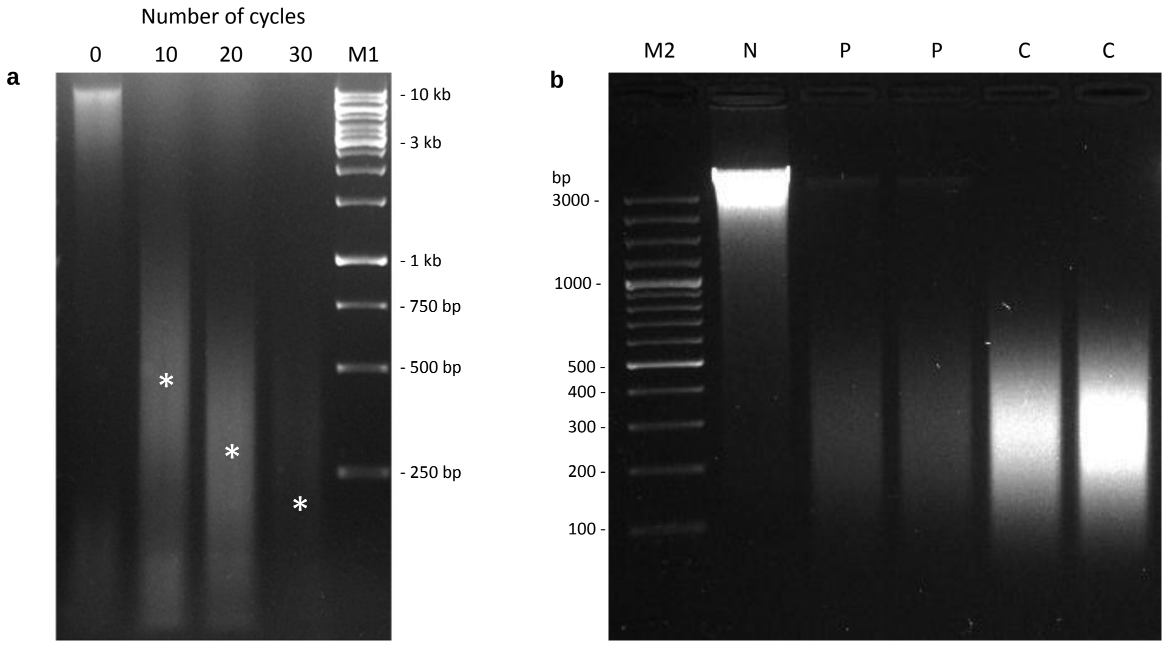

Figure S3. Agarose gel electrophoresis analysis of sonication conditions. (a) Developing secondary xylem chromatin sonicated for various ten-second cycles. Asterisks indicate the average fragment size. M1, GeneRuler 1 kb DNA ladder. **(b)** Analysis of residual DNA in the nuclear pellet following twenty cycles of sonication. N, unsonicated nuclear pellet; P, residual nuclear pellet after sonication (two extractions); C, chromatin after sonication M2, GeneRuler 100 bp DNA ladder plus.

a

b

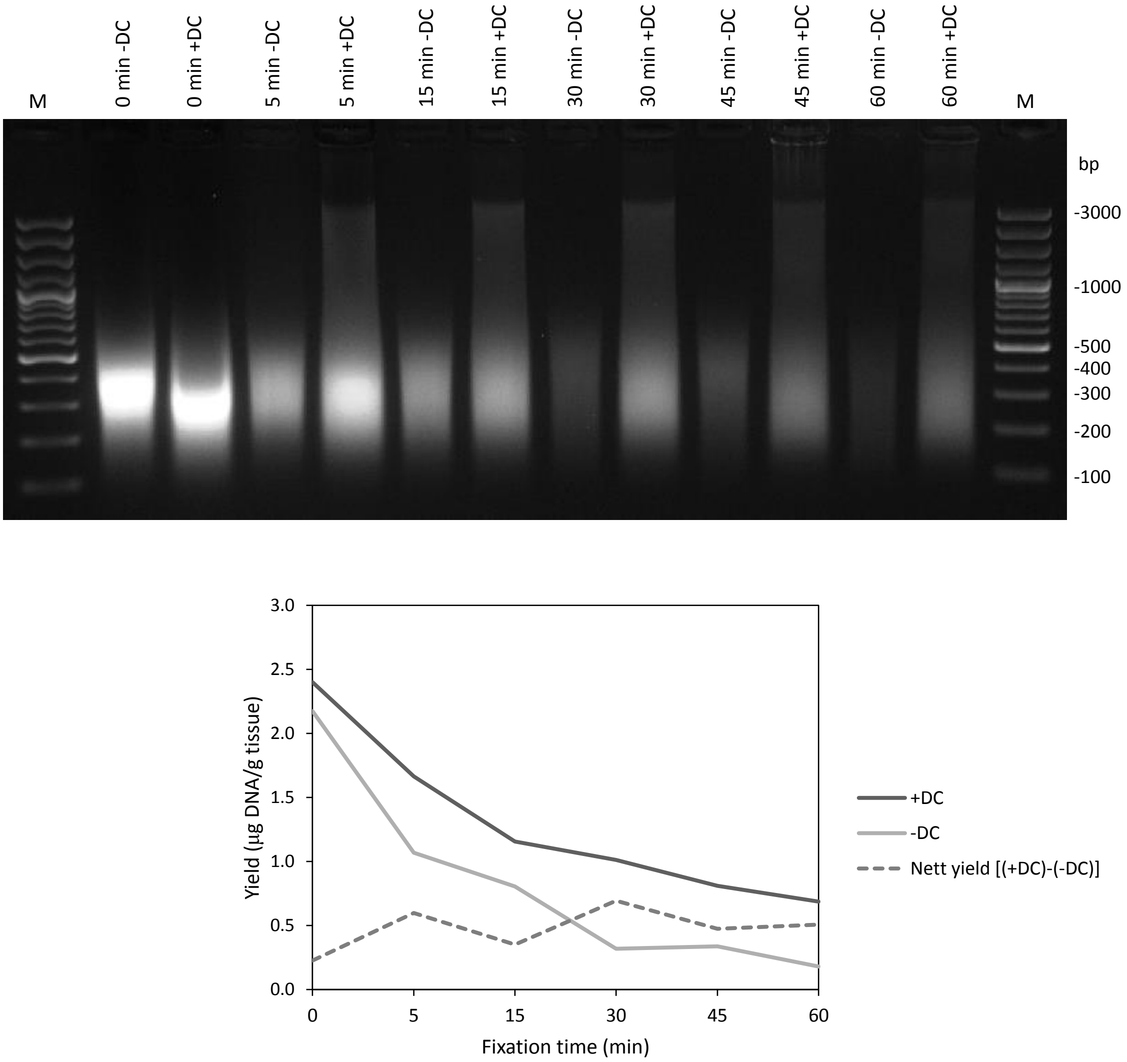

Figure S4. Optimization of formaldehyde-mediated crosslinking of *E. grandis* developing secondary xylem samples. (a) Agarose gel electrophoresis analysis of DNA yield from samples crosslinked for 0, 5, 15, 30, 45 or 60 min and subjected to de-crosslinking (+DC) or no de-crosslinking (–DC). M, GeneRuler 100 bp DNA ladder plus. **(b)** Nett yield of DNA between de-crosslinked (+DC) samples and samples without de-crosslinking treatment (-DC). Samples fixed for 5 and 15 minutes show poor crosslink retention as measured by the ability to extract DNA without prior de-crosslinking, while more than 30 minutes of fixation led to compromised DNA yield after de-crosslinking.

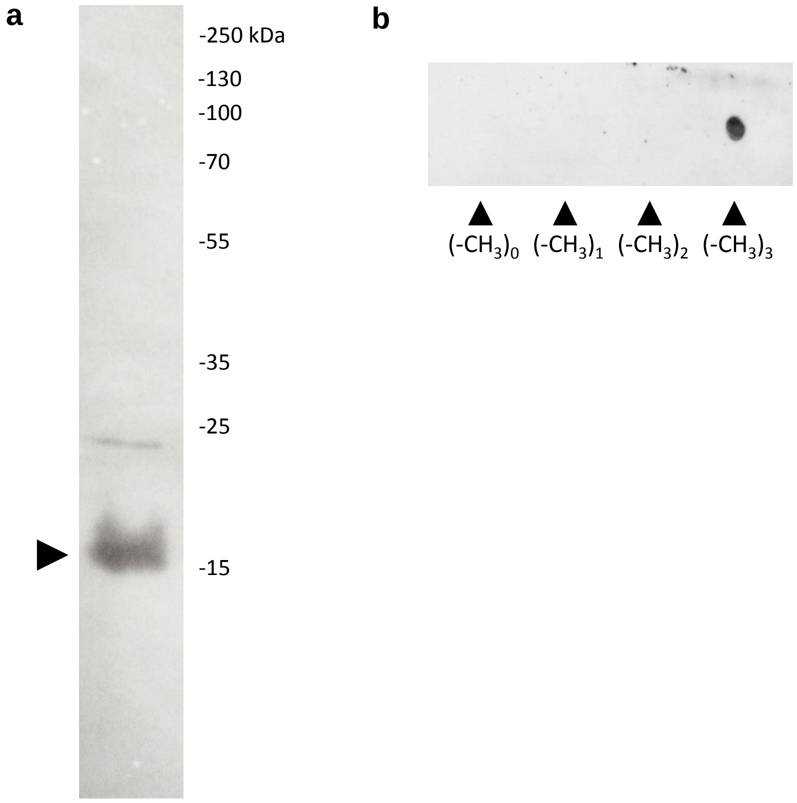

Figure S5. Validation of anti-H3K4me3 antibody specificity. (a) Western blot analysis of *E. grandis* developing secondary xylem nuclear protein extracts using anti-H3K4me3 antibody. The 17 kDa target protein (arrows) comprises over 50% of the lane signal and thus passes ENCODE requirements [5]. **(b)** Dotblot of synthetic peptides (100 ng, sequence NH_2-MART<u>K</u>QTAR-COOH) representing non-methylated histone H3K4 [$(-CH_3)_0$] and mono-, di- or tri-methylated lysine-4 residues [$(-CH_3)_1$, $(-CH_3)_2$ and $(-CH_3)_3$, respectively] using anti-H3K4me3 antibody.

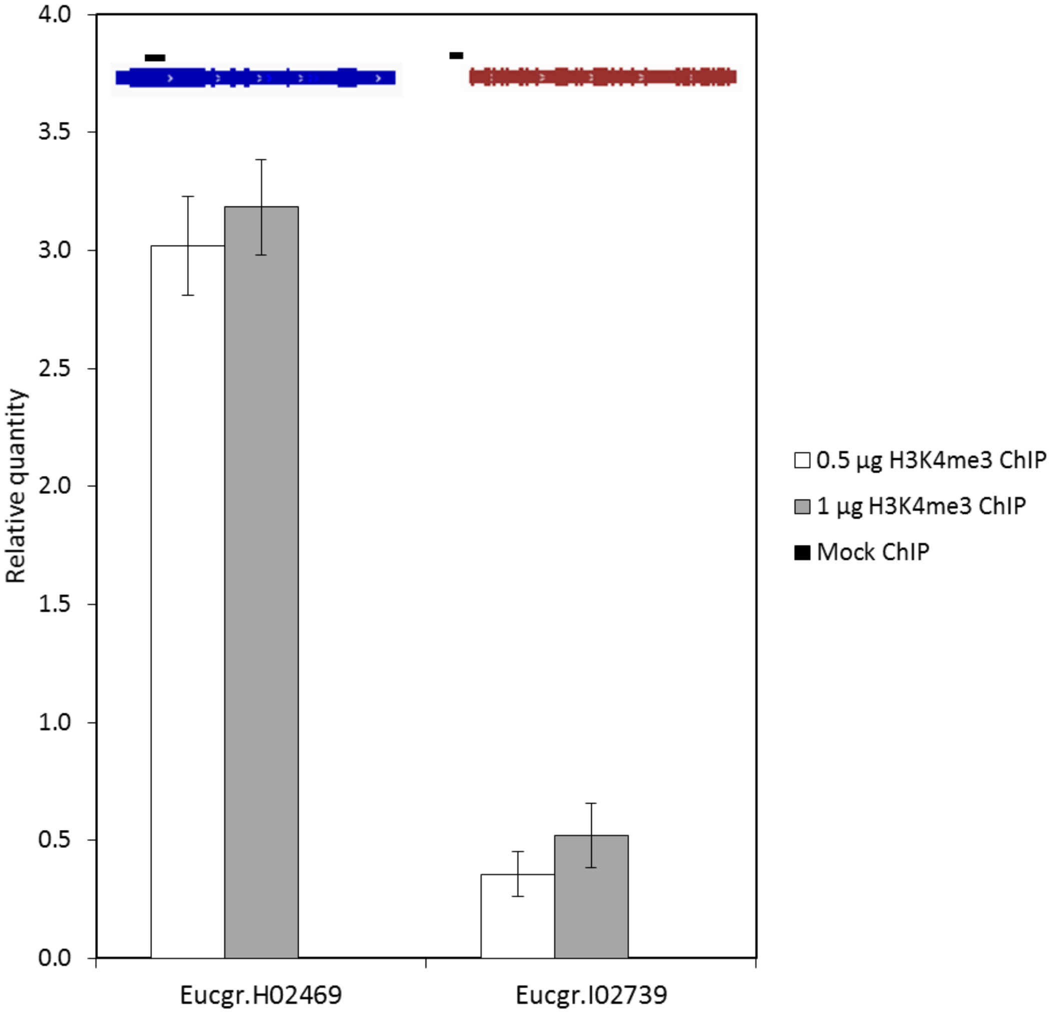

Figure S6. ChIP-qPCR analysis of two candidate loci using different quantities of anti-H3K4me3 antibody. Values are expressed relative to input, where an equal quantity of template was used for qPCR analysis from each sample. Error bars indicate standard deviation of three technical replicates. The targeted regions are shown relative to gene models (blue) with a back bar. Primers are listed for each gene in Additional file 3, Table S7.

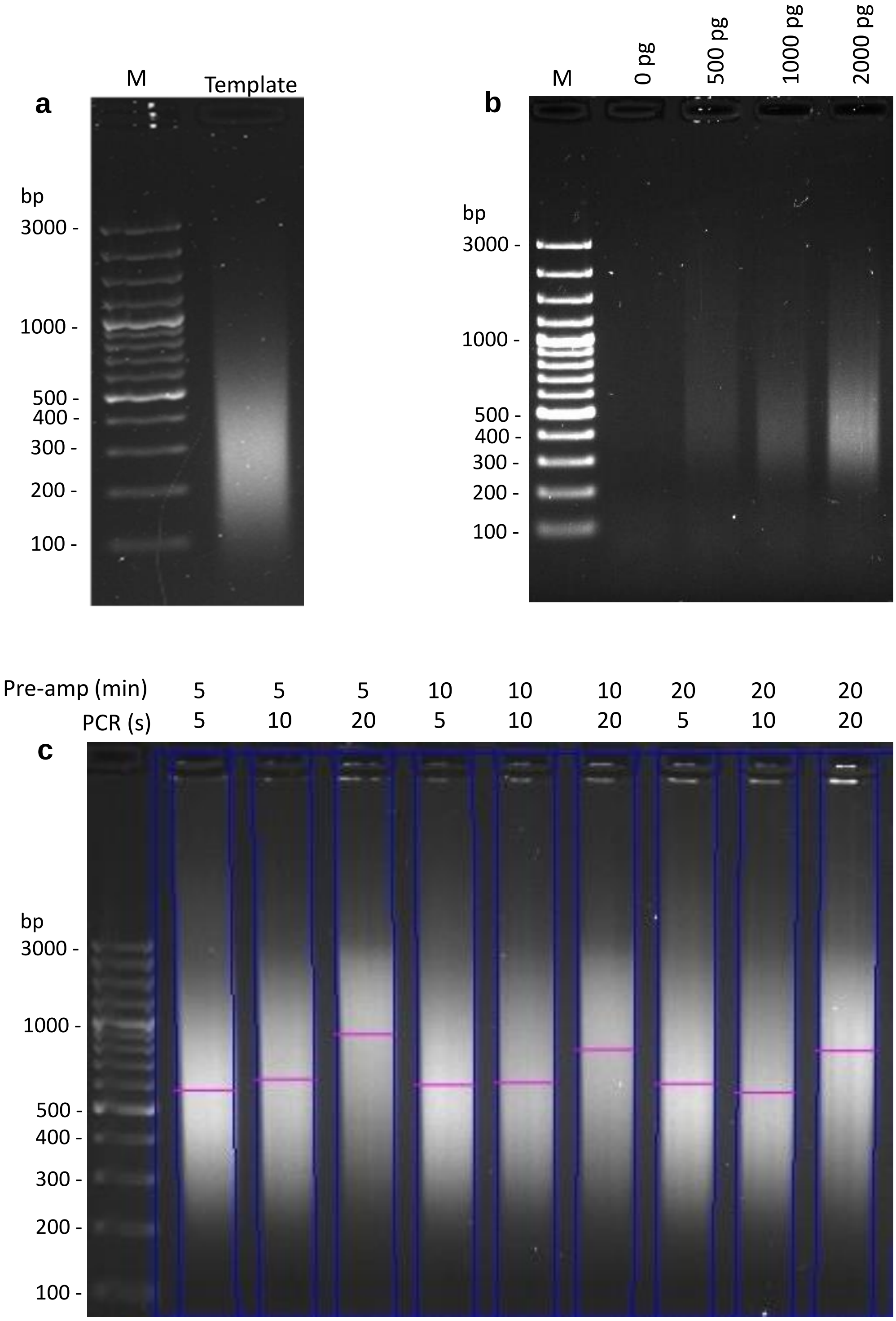

Figure S7. Optimization of ChIP DNA amplification. (a) Fragment length distribution of concentrated template DNA. **(b)** Fragment length distribution of amplified DNA (15 cycles) starting from various template quantities. **(c)** Agarose gel electrophoresis analysis of amplified DNA fragment length using various pre-amplification and PCR extension times (seconds). 20 min pre-amplification and 5 s extension times were used for ChIP DNA amplification (see Methods). M, GeneRuler 100 bp DNA ladder plus marker.

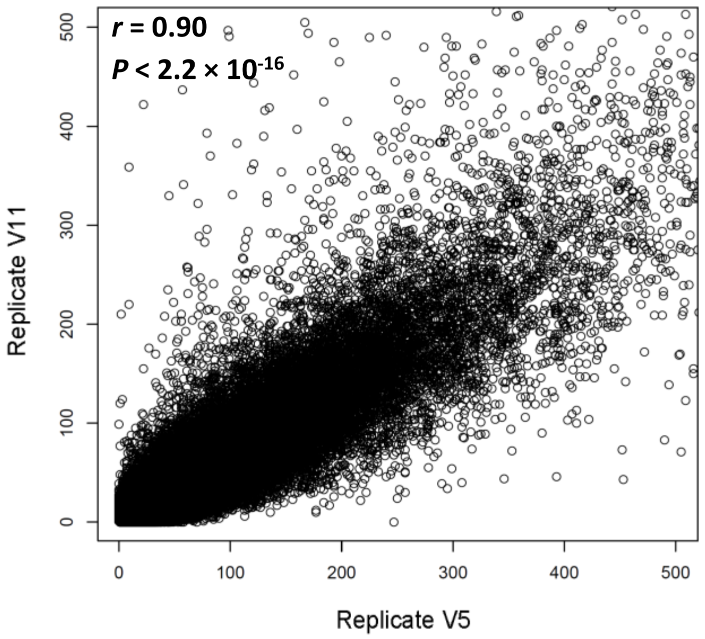

Figure S8. Scatter plot of number of mapped reads of H3K4me3 libraries at 1 kb intervals across the genome between biological replicates V5 and V11.

a

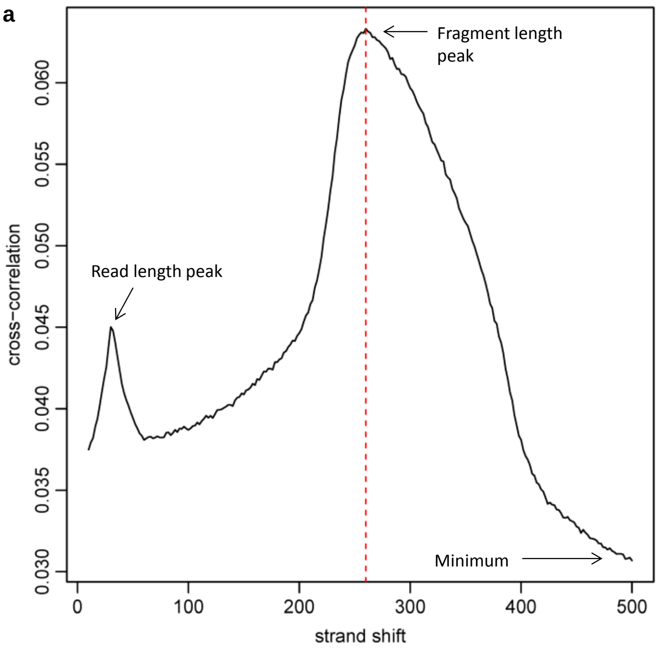

b

$$NSC = \frac{cc(\text{fragment length})}{cc(\text{min})}$$

$$RSC = \frac{cc(\text{fragment length}) - cc(\text{min})}{cc(\text{read length}) - cc(\text{min})}$$

c

	NSC (1.05)	RSC (0.80)
V5 H3K4me3 (left reads)	2.06	2.28
V5 H3K4me3 (right reads)	2.07	2.29
V11 H3K4me3 (left reads)	2.18	1.72
V11 H3K4me3 (right reads)	2.18	1.60

Figure S9. Strand cross-correlation (cc) analysis [5] of mapped ChIP and input library reads. (a) H3K4me3 library (left reads) of individual V5 as representative of ChIP library samples, showing peaks corresponding to read length and fragment length. **(b)** Formulae for calculating normalized strand cross-correlation (NSC) and relative strand cross-correlation (RSC) values. **(c)** NSC and RSC values for all ChIP-seq samples, showing the minimum threshold preferred by ENCODE [5] in parentheses. All H3K4me3 ChIP libraries yielded NSC and RSC values well above 1.05 and 0.8, respectively. Left and right reads were analyzed separately because paired-end reads yield high RSC values by default.

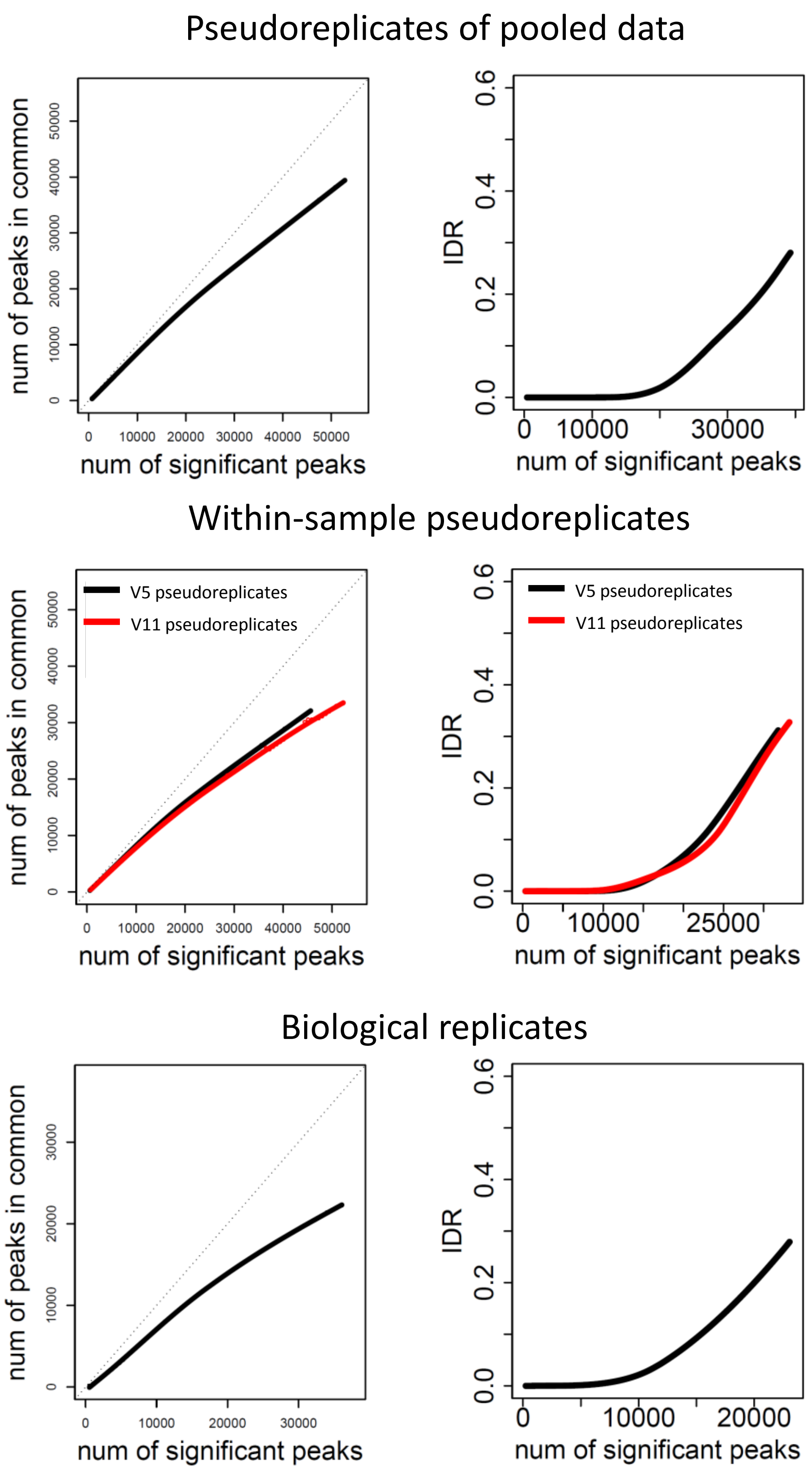

Figure S10. Consistency of peaks identified in pseudoreplicates of pooled reads, pseudoreplicates of each biological replicate (self pseudoreplicates), and biological replicates V5 and V11. The relationship between the number of peaks shared between datasets for an increasing number of identified peaks is shown in the left hand panel; the

right hand panel illustrates the relationship between the irreproducible discovery rate (IDR) and the number of significant peaks identified.

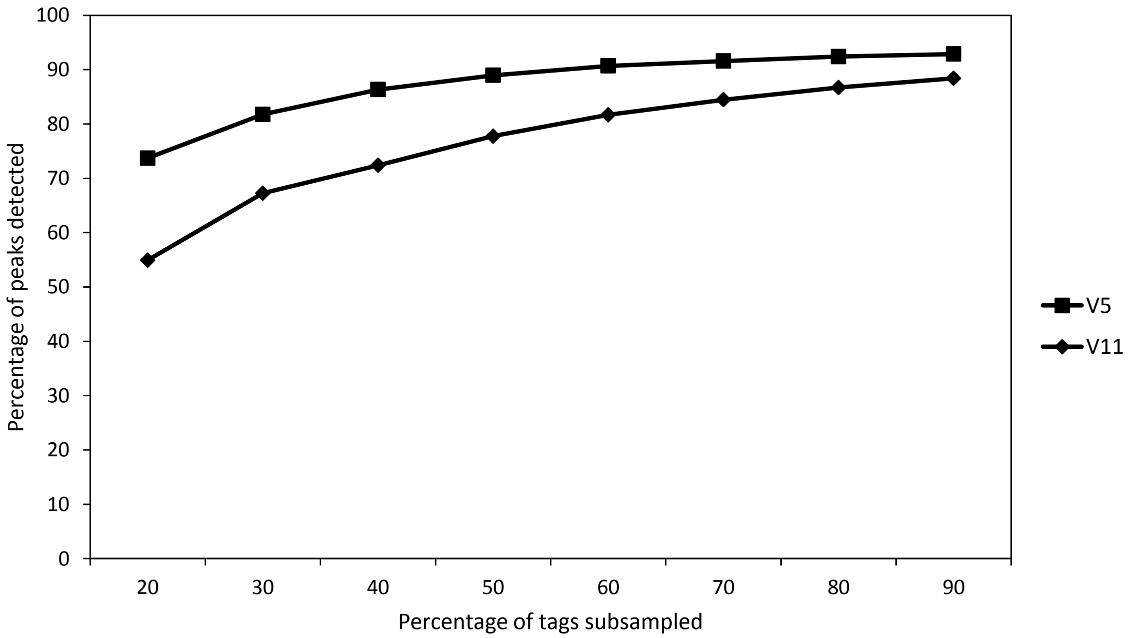

Figure S11. MACS diagnostic analysis of H3K4me3 peaks (fold enrichment 5 - 15) detected for increasing proportions of subsampled mapped sequence tags. The percentage of peaks detected using all tags (*y*-axis) as a function of the proportion of the total tags used for peak detection (*x*-axis) is shown separately for individual V5 and V11.

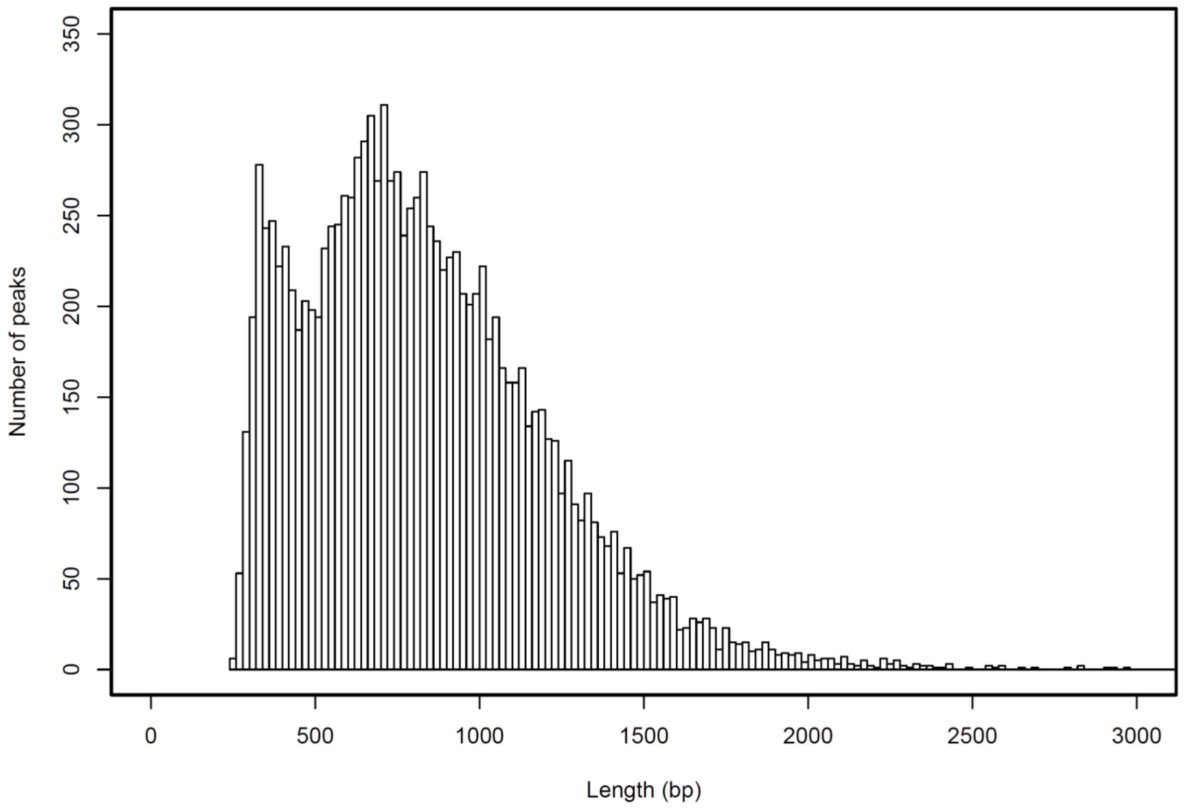

Figure S12. Histogram showing length distribution (bp) of significant H3K4me3 peaks.

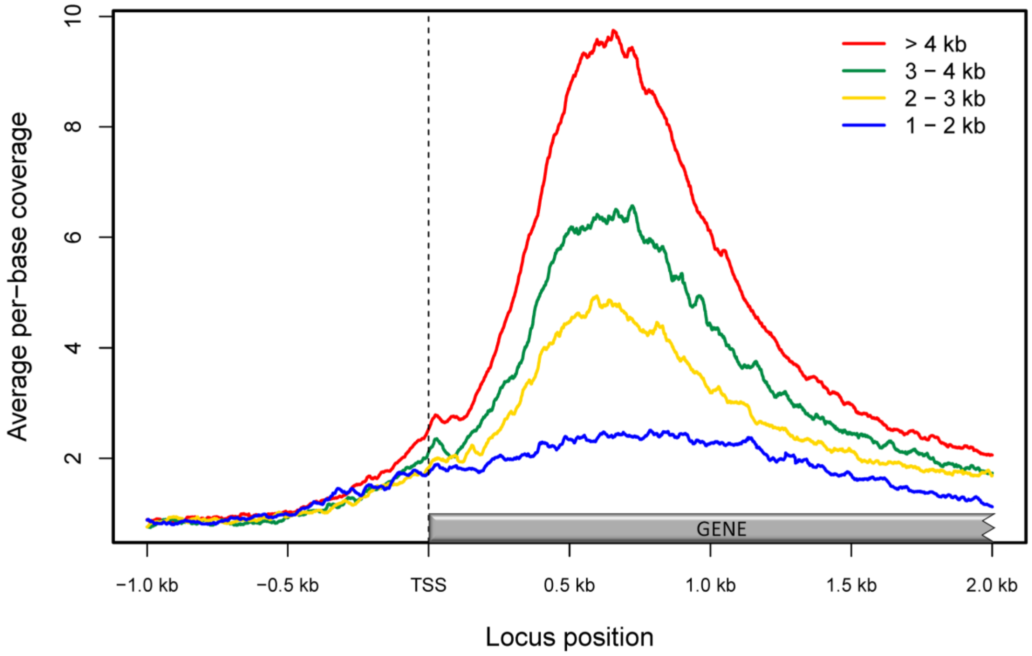

Figure S13. Average H3K4me3 ChIP-seq library per-base read coverage across 5' gene regions of genes of various lengths. TSS, transcription start site.

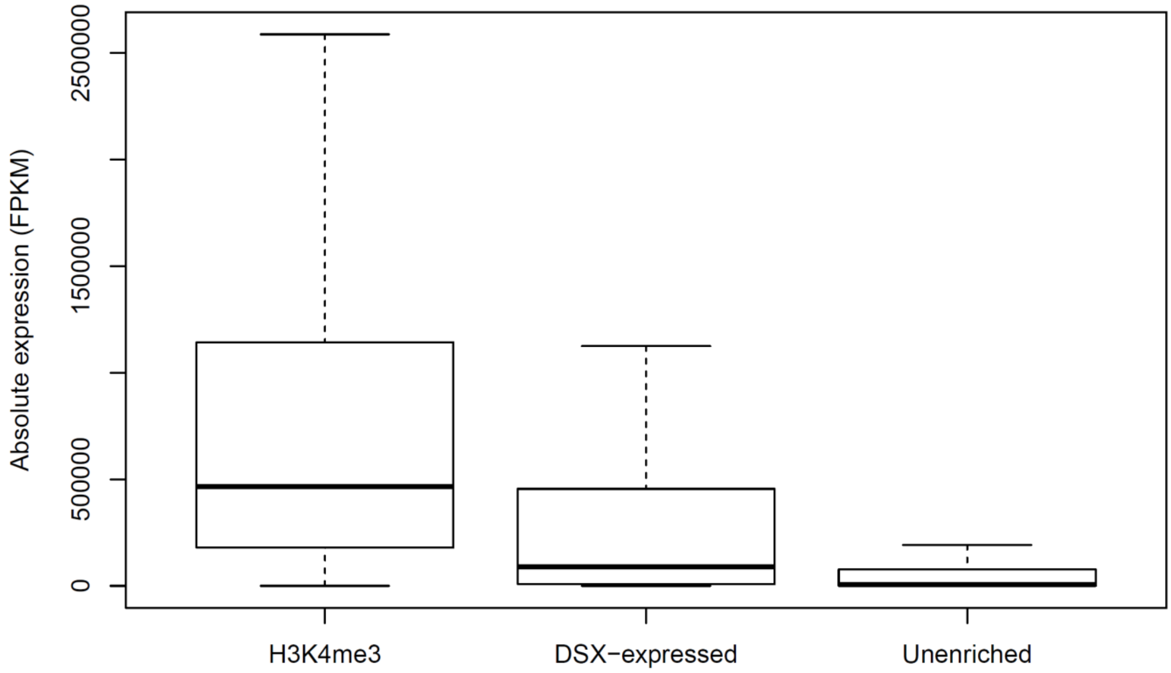

Figure S14. Boxplot of absolute expression levels for H3K4me3-enriched and unenriched genes. Median FPKM (fragments per kilobase per million fragments mapped) values for genes trimethylated at H3K4 (H3K4me3; n = 9,623), genes expressed in developing secondary xylem (DSX-expressed; n = 27,595), or genes lacking H3K4me3 (Unenriched; n = 23,760) are indicated by the central bar. Outliers are not shown.

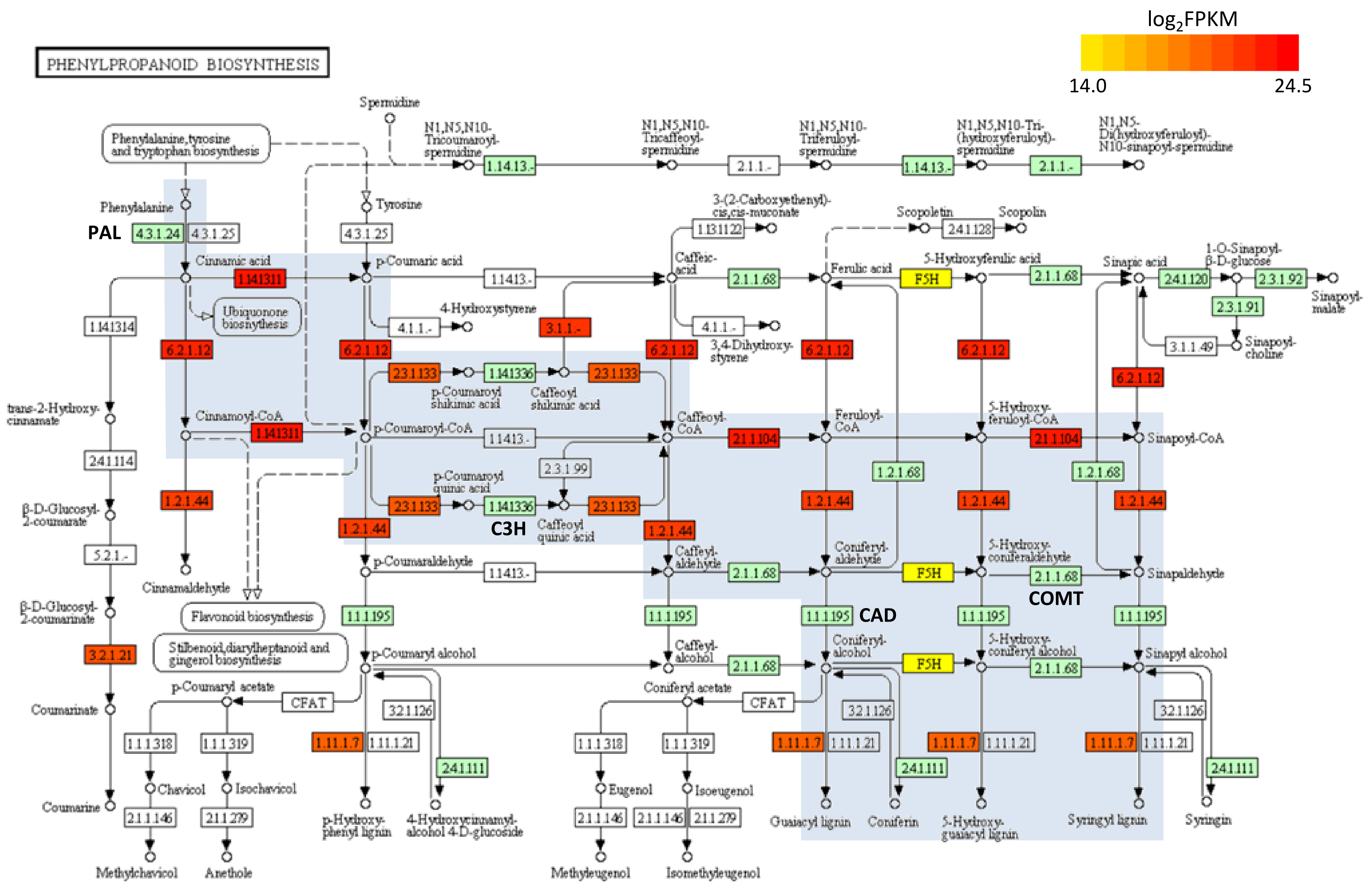

PHENYLPROPANOID BIOSYNTHESIS
log2FPKM
14.0
24.5
PAL
C3H
CAD
COMT
Phenylalanine, tyrosine and tryptophan biosynthesis
Phenylalanine
Tyrosine
Spermidine
Cinnamic acid
p-Coumaric acid
Caffeic acid
Ferulic acid
5-Hydroxyferulic acid
Sinapic acid
Cinnamoyl-CoA
p-Coumaroyl-CoA
Caffeoyl-CoA
Feruloyl-CoA
5-Hydroxy-feruloyl-CoA
Sinapoyl-CoA
p-Coumaraldehyde
Caffeyl-aldehyde
Coniferyl-aldehyde
5-Hydroxy-coniferaldehyde
Sinapaldehyde
p-Coumaryl alcohol
Caffeyl-alcohol
Coniferyl-alcohol
5-Hydroxy-coniferyl alcohol
Sinapyl alcohol
p-Hydroxy-phenyl lignin
Guaiacyl lignin
5-Hydroxy-guaiacyl lignin
Syringyl lignin
Coniferin
Syringin
Scopoletin
Scopolin
Flavonoid biosynthesis
Stilbenoid, diarylheptanoid and gingerol biosynthesis
Ubiquinone biosynthesis
Coumarinate
Coumarine
Cinnamaldehyde
Eugenol
Isoeugenol
Chavicol
Isochavicol
Methylchavicol
Anethole
Methyleugenol
Isomethyleugenol
p-Coumaryl acetate
Coniferyl acetate
CFAT
F5H

Figure S15 ***(previous page).*** H3K4me3-mediated regulation of the phenylpropanoid biosynthetic pathway in *E. grandis*. Expression values (developing secondary xylem tissue) [6] of *Arabidopsis* homologs of *E. grandis* genes enriched for H3K4me3 are shown by the heat map. Green boxes denote *Arabidopsis* enzymes not enriched for H3K4me3 in *E. grandis*; white boxes indicate automatically assigned Enzyme Commission (EC) numbers not represented by any known protein in *Arabidopsis*. Blue shading highlights the principal pathway for monolignol biosynthesis.

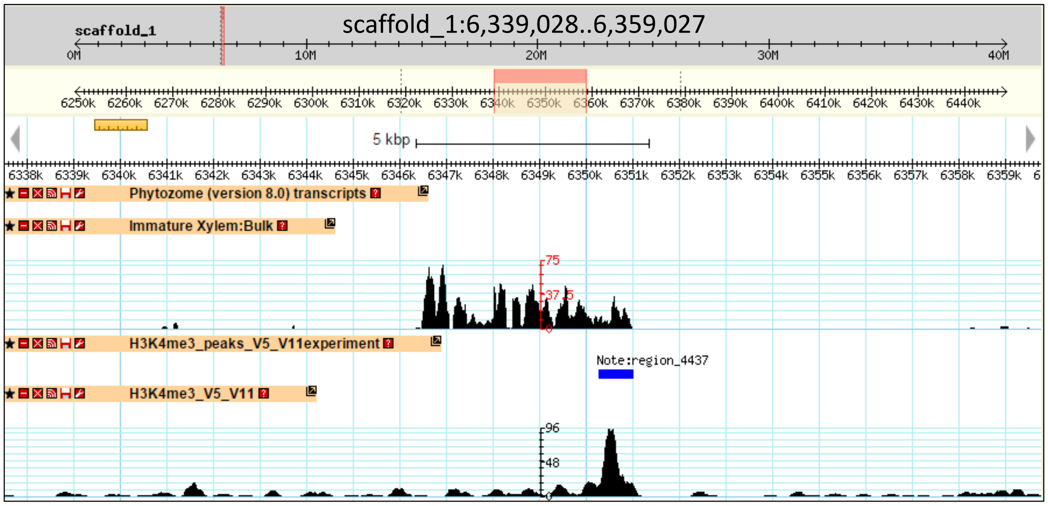
scaffold_1:6,339,028..6,359,027
scaffold_1
5 kbp
Phytozome (version 8.0) transcripts
Immature Xylem:Bulk
75
37.5
0
H3K4me3_peaks_V5_V11experiment
Note:region_4437
H3K4me3_V5_V11
96
48
0

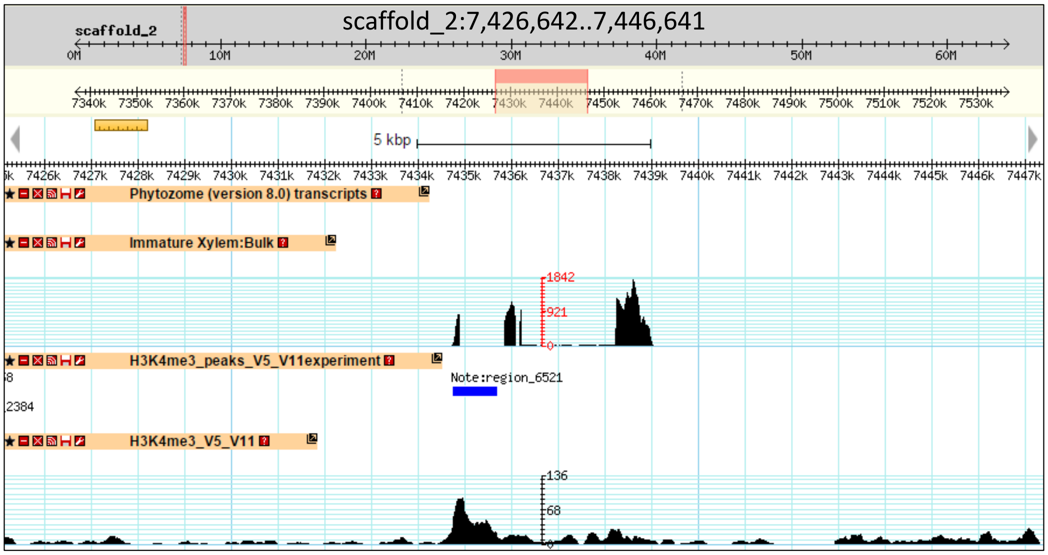
scaffold_2:7,426,642..7,446,641
scaffold_2
5 kbp
Phytozome (version 8.0) transcripts
Immature Xylem:Bulk
1842
921
0
H3K4me3_peaks_V5_V11experiment
Note:region_6521
H3K4me3_V5_V11
136
68
0

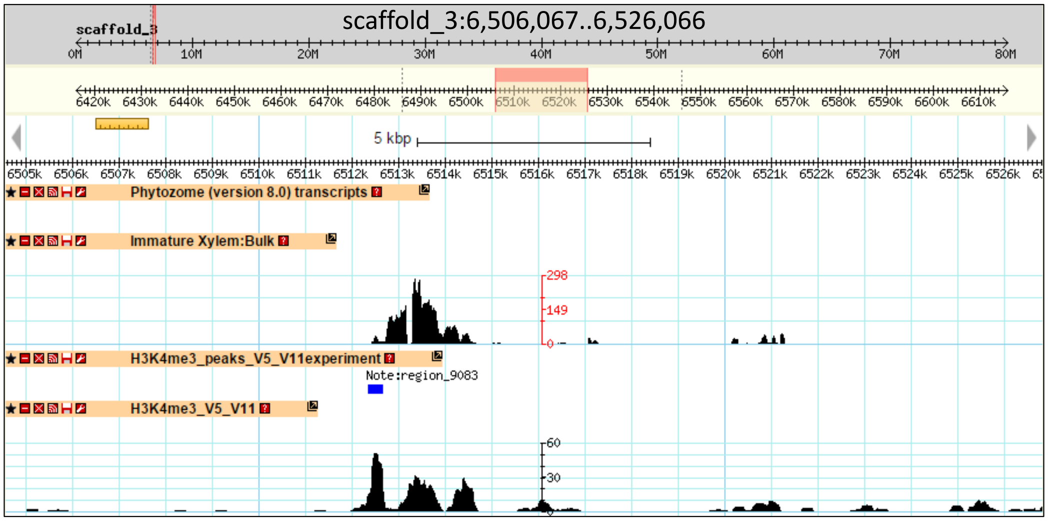
scaffold_3:6,506,067..6,526,066
scaffold_3
5 kbp
Phytozome (version 8.0) transcripts
Immature Xylem:Bulk
298
149
0
H3K4me3_peaks_V5_V11experiment
Note:region_9083
H3K4me3_V5_V11
60
30
0

Figure S16. Three examples of H3K4me3 peaks overlapping transcribed regions that have not been annotated. RNA-seq coverage for developing secondary xylem (here referred to as "immature xylem") are indicated by the track "Immature Xylem: Bulk". H3K4me3 peaks are indicated by blue bars in the "H3K4me3_peaks_V5_V11experiment" track. The "H3K4me3_V5_V11" track denotes the ChIP-seq signal. Each window is 20 kb. Results were visualized in the EucGenIE browser [6].

References

1. Loureiro J, Rodriguez E, Doležel J, Santos C: **Comparison of four nuclear isolation buffers for plant DNA flow cytometry**. *Annals of Botany* 2006, **98**:679–689.
2. Kaufmann K, Muiño J, Østerås M, Farinelli L, Krajewski P, Angenent GC: **Chromatin immunoprecipitation (ChIP) of plant transcription factors followed by sequencing (ChIP-SEQ) or hybridization to whole genome arrays (ChIP-CHIP)**. *Nature Protocols* 2010, **5**:457–472.
3. Saleh A, Alvarez-Venegas R, Avramova Z: **An efficient chromatin immunoprecipitation (ChIP) protocol for studying histone modifications in *Arabidopsis* plants**. *Nature Protocols* 2008, **3**(6):1018-1025.
4. McKeown P, Pendle AF, Shaw PJ: **Preparation of *Arabidopsis* nuclei and nucleoli**. In: *The Nucleus: Nuclei and Subnuclear Components.* Humana Press; 2008: 65-74.
5. Landt SG, Marinov GK, Kundaje A, Kheradpour P, Pauli F, Batzoglou S, Bernstein BE, Bickel P, Brown JB, Cayting P *et al*: **ChIP-seq guidelines and practices of the ENCODE and modENCODE consortia**. *Genome Research* 2012, **22**:1813-1831.
6. Hefer C, Mizrachi E, Joubert F, Myburg A: **The *Eucalyptus* genome integrative explorer (EucGenIE): a resource for *Eucalyptus* genomics and transcriptomics**. *BMC Proceedings* 2011, **5**(Suppl 7):O49.
