# Supplementary material for: Genome-wide mapping of histone H3 lysine 4 trimethylation in Eucalyptus grandis developing xylem
Source: BMC Plant Biol. 2015 May 10;15:117. doi: 10.1186/s12870-015-0499-0 (PMC4425858; doi:10.1186/s12870-015-0499-0)
Supplement: Additional file 3: — Table S1, Table S2, Table S3, Table S4, Table S5, Table S6, Table S7. [file 12870_2015_499_MOESM3_ESM.pdf]

Supplementary tables

Table S1. ChIP-seq library sequence and mapping statistics. Prefixes V5 and V11 refer to the two individuals sampled.

Dataset	Read pairs after filtering	Read length after trimming	Read pairs uniquely mapped	% uniquely mapped
V5_Input	20,846,731	31 nt	11,464,953	55.0%
V5_H3K4me3	21,907,313	31 nt	5,707,963	26.1%
V11_Input	11,939,885	35 nt	6,709,264	56.2%
V11_H3K4me3	11,868,984	35 nt	3,741,400	31.5%
V11_IgG_{2a}	11,692,593	35 nt	1,144,387	9.8%

Table S2. Number of significant peaks shared between biological replicates and randomly subsampled pseudoreplicates

Dataset comparison	IDR threshold*	Number of shared peaks
V5 vs V11 biological replicates	0.02	9835
V5 within-sample pseudoreplicates	0.02	15202
V11 within-sample pseudoreplicates	0.02	14584
Pseudoreplicates of pooled data	0.01	18333

*IDR, Irreproducible Discovery Rate. Thresholds, which vary depending on the context, were selected based on guidelines provided by the software developers (https://sites.google.com/site/anshulkundaje/projects/idr#TOC-IDR-PIPELINE).

Table S3. Putative targets of H3K4me3-regulated miRNAs in Table 1. Targets were identified using psRNATarget [1].

miRNA locus	Putative target transcript	FPKM (DSX)*	FPKM (max)†	Description‡
scaffold_3:4901554..4901642	Eucgr.A02249	251 802	1 607 587	Glutamate synthase 1
	Eucgr.G00560	9 433	481 637	Potassium transport 2/3
	Eucgr.G03334	224 530	477 999	Structural maintenance of chromosomes 5
	Eucgr.K01811	177 715	240 500	Nuclear RNA polymerase A1
scaffold_7:40119641..40119697	Eucgr.A02400	124 136	355 397	C2H2-type zinc finger family protein
	Eucgr.E04257	9 893	162 788	Ankyrin repeat family protein
	Eucgr.E04270	596	12 859	Ankyrin repeat family protein
	Eucgr.K03411	172	29 119	Auxin-responsive GH3 family protein
	Eucgr.B03816	588 151	665 806	Transcription factor-related
	Eucgr.H00927	278 873	413 977	S-adenosyl-L-methionine-dependent methyltransferases superfamily protein
scaffold_7:40120411..40120431	Eucgr.D00906	104	11 567	S-locus lectin protein kinase family protein
	Eucgr.D00895	0	8 487	S-locus lectin protein kinase family protein
	Eucgr.H00013	0	984	O-acyltransferase (WSD1-like) family protein
	Eucgr.C01384	151 500	225 507	Small nuclear RNA activating complex (SNAPc), subunit SNAP43 protein
	Eucgr.C02330	2 664 123	3 219 047	P450 reductase 2
	Eucgr.F03969	283 580	333 211	Magnesium transporter 2
scaffold_8:3505820..3505839	Eucgr.C01226	438	14 488	Protein of unknown function (DUF594)
	Eucgr.C01222	0	16 690	Protein of unknown function (DUF594)
	Eucgr.C01227	1 137	15 738	Protein of unknown function (DUF594)
	Eucgr.C01223	2 992	17 172	Protein of unknown function (DUF594)
	Eucgr.C01221	182	5 890	Protein of unknown function (DUF594)
	Eucgr.C01219	506	20 039	Protein of unknown function (DUF594)
	Eucgr.A00874	51 102	112 657	F-box/RNI-like superfamily protein
	Eucgr.A00876	119 103	171 048	RNI-like superfamily protein
scaffold_9:28910581..28910654	Eucgr.A02961	160 937	333 983	Protein kinase family protein with ARM repeat domain
	Eucgr.K03271	17 048	267 828	2-oxoglutarate (2OG) and Fe(II)-dependent oxygenase superfamily protein
	Eucgr.K03275	56 413	217 741	2-oxoglutarate (2OG) and Fe(II)-dependent oxygenase superfamily protein
scaffold_11:43275663..43275749	Eucgr.C01693	220 239	624 348	Disease resistance protein (TIR-NBS-LRR class), putative
	Eucgr.E01103	11 802	58 082	Cinnamyl-alcohol dehydrogenase
	Eucgr.A01774	218 633	3 241 210	Photosystem II reaction center PSB29 protein
	Eucgr.B01005	716 973	993 092	NAD(P)-linked oxidoreductase superfamily protein
	Eucgr.K01262	620 431	620 431	SCAR homolog 2
	Eucgr.G02722	511 932	760 518	Protein of unknown function DUF455
	Eucgr.E00455	0	746	Flavin-binding monooxygenase family protein
	Eucgr.A00534	295 577	538 206	AMP deaminase, putative / myoadenylate deaminase, putative
	Eucgr.E01036	95 389	174 242	Tetratricopeptide repeat (TPR)-like superfamily protein
	Eucgr.F02840	4 013 037	9 666 673	Calmodulin like 23
	Eucgr.K01542	1 183	277 272	Myb domain protein 56
	Eucgr.F00956	107 773	289 943	NB-ARC domain-containing disease resistance protein

*RNA-seq data for developing secondary xylem (DSX) was obtained from EucGenIE [2]
†Maximum transcript abundance observed across seven tissues in the EucGenIE database
‡*E. grandis* annotation v.1.1 (www.phytozome.net)

Table S4. Significantly overrepresented and underrepresented biological functions among H3K4-trimethylated genes

GO identifier	Level(s)	Term	Frequency (set)	Frequency (reference)	*P*-value*
**Enriched**					
GO:0044267	6,5	cellular protein metabolic process	0.1082	0.0842	1.68E-19
GO:0006412	7,5,6	translation	0.0391	0.0272	4.31E-14
GO:0019538	5,4	protein metabolic process	0.1235	0.1016	2.74E-13
GO:0044257	7,6	cellular protein catabolic process	0.0175	0.0113	8.99E-09
GO:0043632	6	modification-dependent macromolecule catabolic process	0.0166	0.0106	9.16E-09
GO:0006511	10,8,9	ubiquitin-dependent protein catabolic process	0.0166	0.0106	9.16E-09
GO:0019941	9,7,8	modification-dependent protein catabolic process	0.0166	0.0106	9.16E-09
GO:0051603	8,7	proteolysis involved in cellular protein catabolic process	0.0169	0.0109	1.15E-08
GO:0044260	4	cellular macromolecule metabolic process	0.2144	0.1929	4.84E-07
GO:0034960	5	cellular biopolymer metabolic process	0.2096	0.189	1.72E-06
GO:0034962	6	cellular biopolymer catabolic process	0.02	0.0139	3.59E-06
GO:0044265	5	cellular macromolecule catabolic process	0.0248	0.018	3.96E-06
GO:0043170	3	macromolecule metabolic process	0.2337	0.2144	4.92E-05
GO:0043283	4	biopolymer metabolic process	0.2254	0.2075	0.000245
GO:0006996	3	organelle organization	0.0402	0.0322	0.000278
GO:0009987	2	cellular process	0.4105	0.3897	0.000475
GO:0051649	4,3	establishment of localization in cell	0.031	0.0244	0.001192
GO:0046907	5,4	intracellular transport	0.0272	0.0211	0.001658
GO:0016192	3,4,5	vesicle-mediated transport	0.0126	0.0087	0.001686
GO:0044238	3	primary metabolic process	0.2983	0.2804	0.002255
GO:0008104	4	protein localization	0.022	0.0167	0.002852
GO:0044248	4	cellular catabolic process	0.0371	0.0302	0.004243
GO:0045184	3,4,5	establishment of protein localization	0.0205	0.0156	0.006344
GO:0015031	4,5,6	protein transport	0.0205	0.0156	0.006344
GO:0006399	8,7	tRNA metabolic process	0.0083	0.0054	0.010812
GO:0051641	3	cellular localization	0.0328	0.0267	0.013586
GO:0016071	7,6	mRNA metabolic process	0.0077	0.005	0.014605
GO:0043038	6,7	amino acid activation	0.0061	0.0039	0.032366
GO:0043039	7,9,8	tRNA aminoacylation	0.0061	0.0039	0.032366
GO:0006418	8,10,9,6,7	tRNA aminoacylation for protein translation	0.0061	0.0039	0.032366
GO:0006066	4	cellular alcohol metabolic process	0.0131	0.0096	0.034377
GO:0044237	3	cellular metabolic process	0.3046	0.2895	0.042549
**Depleted**					
GO:0006952	4	defense response	0.0191	0.0324	7.58E-14
GO:0050896	2	response to stimulus	0.1462	0.1745	8.30E-14
GO:0019748	3	secondary metabolic process	0.0099	0.0197	3.74E-12
GO:0042221	3	response to chemical stimulus	0.0681	0.0822	6.65E-06
GO:0015979	4	photosynthesis	0.0049	0.01	1.30E-05
GO:0006355	8,7,9,6	regulation of transcription, DNA-dependent	0.0245	0.0333	4.23E-05
GO:0051252	7,6,5	regulation of RNA metabolic process	0.0248	0.0335	7.86E-05
GO:0009699	7,6,5	phenylpropanoid biosynthetic process	0.0032	0.0073	0.00014
GO:0009698	6,5,4	phenylpropanoid metabolic process	0.0043	0.0087	0.00021
GO:0006725	4	cellular aromatic compound metabolic process	0.0124	0.0186	0.00042
GO:0032774	5,7,6	RNA biosynthetic process	0.027	0.0351	0.00082
GO:0006351	8,6,7	transcription, DNA-dependent	0.027	0.0351	0.00082
GO:0065007	2	biological regulation	0.148	0.1636	0.000887
GO:0042398	6,5	cellular amino acid derivative biosynthetic process	0.0061	0.0106	0.00159
GO:0009719	3	response to endogenous stimulus	0.0403	0.0495	0.001613
GO:0006950	3	response to stress	0.0853	0.0977	0.001633
GO:0050794	4,3	regulation of cellular process	0.112	0.1253	0.002735
GO:0045449	7,8,6	regulation of transcription	0.054	0.0638	0.004199
GO:0010200	6	response to chitin	0.0041	0.0078	0.004747
GO:0031326	6,5	regulation of cellular biosynthetic process	0.0578	0.0677	0.005027
GO:0009889	5,4	regulation of biosynthetic process	0.0578	0.0677	0.005027
GO:0019219	6,5	regulation of nucleobase, nucleoside, nucleotide and nucleic acid metabolic process	0.0565	0.0663	0.006397

GO:0010556	6,5	regulation of macromolecule biosynthetic process	0.056	0.0657	0.006573
GO:0009813	8,7,6	flavonoid biosynthetic process	0.0014	0.0039	0.012393
GO:0019684	5	photosynthesis, light reaction	0.0034	0.0065	0.024446
GO:0019438	5	aromatic compound biosynthetic process	0.0068	0.0108	0.025312
GO:0080090	5,4	regulation of primary metabolic process	0.0616	0.0708	0.025989
GO:0009753	4	response to jasmonic acid stimulus	0.0052	0.0087	0.03729
GO:0006575	5	cellular amino acid derivative metabolic process	0.0094	0.0136	0.040039
GO:0031323	5,4	regulation of cellular metabolic process	0.0663	0.0754	0.042139
GO:0050789	3,2	regulation of biological process	0.1275	0.1392	0.044805
GO:0006350	7,5	transcription	0.0592	0.0679	0.048128

*Bonferroni correction

Table S5. H3K4me3 enrichment of *E. grandis* genes involved in secondary cell wall polysaccharide biosynthesis. Gene lists obtained from Myburg *et al.* [3].

Gene model	H3K4me3-enriched?	*Arabidopsis* BLAST hit	*Arabidopsis* gene name	Relative expression (%)*	Absolute expression (FPKM)†
**SUSY**					
Eucgr.C03199	√	AT3G43190	SUS4	46.1	27 043 300
Eucgr.C00769	√	AT3G43190	SUS4	52.8	6 929 210
Eucgr.H01094	√	AT4G02280	SUS3	13.9	1 947 340
Eucgr.F03879	√	AT1G80070	SUS2	10.2	682 244
Eucgr.C03205		AT3G43190	SUS4	45.0	502 145
Eucgr.D02653	√	AT1G01040	SUS1	17.8	486 588
Eucgr.H03515		AT3G43190	SUS4	65.0	398 406
Eucgr.K00816		AT3G43190	SUS4	50.1	375 515
Eucgr.C03207		AT3G43190	SUS4	11.9	141 733
Eucgr.C03204		AT3G43190	SUS4	39.6	114 469
Eucgr.H03496		AT3G43190	SUS4	51.4	69 192
Eucgr.F03935		AT1G80070	SUS2	10.5	52 735
Eucgr.C03201		AT3G43190	SUS4	27.7	32 633
Eucgr.K03505		AT4G02280	SUS3	28.2	21 892
Eucgr.F01010		AT3G43190	SUS4	20.7	17 515
Eucgr.J01640		AT5G37180	SUS5	2.0	13 788
Eucgr.K02305		AT1G73370	SUS6	1.5	5 678
Eucgr.B01577		AT5G49190	SUS2	7.8	141
**Invertase**					
Eucgr.F02588	√	AT4G09510	CINV2	21.6	1 609 980
Eucgr.D02386		AT4G09510	CINV2	0.9	1 004
**Hexokinase**					
Eucgr.B03711	√	AT1G50460	HKL1	27.4	1 335 271
Eucgr.F01647	√	AT1G47840	HXK3	20.0	759 403
Eucgr.C03728	√	AT4G29130	HXK1	18.7	881 978
Eucgr.J00734	√	AT1G50460	HKL1	7.7	658 748
Eucgr.C00983		AT2G19860	HXK2	22.9	46 104
Eucgr.C00975		AT2G19860	HXK2	19.4	2 269
Eucgr.C00559		AT2G19860	HXK2	16.2	35 175
Eucgr.C00569		AT2G19860	HXK2	10.5	5 672
**Phosphoglucomutase**					
Eucgr.G02157	√	AT1G70730	PGM2	35.6	3 172 777
Eucgr.B02942	√	AT1G23190	PGM3	28.7	2 983 813
Eucgr.J01084	√	AT5G17530		24.9	776 193
Eucgr.K00185	√	AT5G51820	PGM1	13.8	608 346
Eucgr.H01421		AT5G17530		11.2	227 897
Eucgr.D02001		AT5G17530		8.4	140 299
Eucgr.L01855		AT1G70820		10.2	8 987
Eucgr.B02929		AT1G70820		0.5	951
**UGP**					
Eucgr.E04308	√	AT2G35020		65.5	10 125 387
Eucgr.F02905	√	AT5G17310	UGP2	35.7	7 822 697
Eucgr.D01117	√	AT3G03250	UGP1	14.7	440 314
Eucgr.J00183	√	AT3G56040	UGP3	10.1	112 668
**CESA**					
Eucgr.D00476		AT4G18780	CESA8	91.8	25 758 933
Eucgr.A01324	√	AT5G44030	CESA4	91.3	23 436 733
Eucgr.C00246		AT5G17420	CESA7	92.7	20 792 367
Eucgr.I00286	√	AT2G21770	CESA9	12.3	3 558 470
Eucgr.G03380	√	AT5G05170	CESA3	17.6	3 133 070
Eucgr.C02801	√	AT4G32410	CESA1	13.3	3 021 617
Eucgr.F03635	√	AT2G21770	CESA9	10.3	1 356 514
Eucgr.C01769	√	AT4G32410	CESA1	21.0	1 205 310
Eucgr.J01278	√	AT5G05170	CESA3	21.3	1 141 560
Eucgr.F04216	√	AT5G64740	CESA6	83.7	789 956
Eucgr.C01147		AT4G32410	CESA1	13.6	299 713
Eucgr.B03971		AT2G21770	CESA9	12.3	156 770
Eucgr.H00939	√	AT4G32410	CESA1	12.2	136 970

Eucgr.J01639		AT4G32410	CESA1	14.8	121 932
Eucgr.A02372		AT5G05170	CESA3	65.8	115 561
Eucgr.F04212		AT5G64740	CESA6	69.1	107 544
Eucgr.B01562		AT2G21770	CESA9	8.6	50 683
Eucgr.H00646		AT2G21770	CESA9	18.1	42 422
Eucgr.H02200		AT2G21770	CESA9	16.7	35 374
Eucgr.B01532		AT2G21770	CESA9	6.2	24 751
Eucgr.L02402		AT4G32410	CESA1	8.7	12 329
**UGD**					
Eucgr.J01372	√	AT5G15490	UGD3	44.7	33 666 800
Eucgr.K02506		AT5G15490	UGD3	49.2	1 362 214
**UXS**					
Eucgr.J00040	√	AT5G59290	UXS3	61.6	13 448 933
Eucgr.G02921		AT2G28760	UXS6	82.4	9 374 193
Eucgr.H01112	√	AT3G62830	UXS2	15.1	6 087 550
Eucgr.A01221	√	AT3G53520	UXS1	48.8	2 712 707
Eucgr.B00451		AT5G59290	UXS3	50.6	192 504
Eucgr.H03936		AT3G62830	UXS2	10.9	22 404
**IRX9**					
Eucgr.A01172		AT2G37090	IRX9	89.8	9 605 213
Eucgr.F02177	√	AT1G27600	I9H,IRX9-L	54.8	609 183
Eucgr.F00463	√	AT1G27600	I9H,IRX9-L	35.2	494 170
Eucgr.K03214		AT2G37090	IRX9	92.4	433 206
Eucgr.C00584	√	AT1G27600	I9H,IRX9-L	18.6	284 588
**IRX10**					
Eucgr.G01977	√	AT1G27440	IRX10	87.8	11 264 120
Eucgr.K02191	√	AT5G61840.1	IRX10-L	30.3	1 304 447
**PARVUS**					
Eucgr.F01531	√	AT3G50760	GATL2	28.7	4 694 067
Eucgr.I01882		AT3G28340	GATL10	23.7	3 587 020
Eucgr.A00485	√	AT1G19300	PARVUS	19.5	756 525
Eucgr.B03054		AT1G13250	GATL3	12.5	312 388
Eucgr.B02574	√	AT1G70090	GATL9,LGT8	12.8	248 814
Eucgr.L02297		AT3G28340	GATL10	31.8	144 978
Eucgr.H01923	√	AT3G50760	GATL2	5.6	112 741
Eucgr.E04362		AT1G02720	GATL5	0.2	314
Eucgr.E02024		AT1G02720	GATL5	0.2	255
Eucgr.H01534		AT3G06260	GATL4	0.0	0
Eucgr.K03408		AT3G06260	GATL4	0.0	0
**IRX14**					
Eucgr.I00880	√	AT5G67230	IRX14-L	57.0	3 409 737
Eucgr.H02219		AT5G67230	IRX14-L	83.5	1 998 313
**RWA**					
Eucgr.D00335	√	AT2G34410	RWA3	78.4	12 710 067
Eucgr.B03976	√	AT3G06550	RWA2	27.9	2 686 777
**DUF579**					
Eucgr.I02785		AT1G09610	GXM3/1	91.5	21 452 567
Eucgr.H02217		AT5G67210	IRX15-L	87.3	19 256 333
Eucgr.I00888	√	AT5G67210	IRX15-L	66.8	7 135 410
Eucgr.I01644		AT1G67330		91.9	1 889 973
Eucgr.F02961	√	AT1G33800	GXMT1	26.4	1 716 353
Eucgr.H02125		AT3G50220	IRX15	90.5	563 951
Eucgr.I01114	√	AT5G67210	IRX15-L	4.1	239 701
Eucgr.K02723		AT4G24910		52.6	209 995
Eucgr.G01567	√	AT5G67210	IRX15-L	31.8	23 509
**IRX7**					
Eucgr.J00384	√	AT2G28110	FRA8,IRX7	56.2	2 854 840
**IRX8**					
Eucgr.F00995		AT5G54690	GAUT12,IRX8	87.9	5 863 770
**PGSIP**					
Eucgr.F00232	√	AT4G33330	GUX2,PGSIP3	75.5	2 360 753
Eucgr.H04942		AT3G18660	GUX1,PGSIP1	90.5	1 967 787
Eucgr.H04216	√	AT5G18480	PGSIP6	19.7	736 868
Eucgr.F02737	√	AT1G77130	GUX3,PGSIP2	15.4	296 346
Eucgr.F04263		AT1G77130	GUX3,PGSIP2	57.2	67 996
Eucgr.L01540		AT1G54940	PGSIP4	0.0	0

**DUF231**					
Eucgr.J00170		AT2G40320	TBL33	95.0	26 938 300
Eucgr.J00985		AT3G54260	TBL36	34.6	12 160 110
Eucgr.G03092	√	AT2G40320	TBL33	59.4	6 589 177
Eucgr.A00530		AT5G01360	TBL3	94.2	5 761 423
Eucgr.J00196	√	AT3G55990	ESK1,TBL29	78.3	3 219 740
Eucgr.F02742	√	AT1G48880	TBL7	30.1	2 796 013
Eucgr.E00070	√	AT1G01430	TBL25	71.4	2 027 880
Eucgr.K02974		AT5G01360	TBL3	81.3	1 785 827
Eucgr.F01211		AT1G73140	TBL31	89.0	1 360 880
Eucgr.C03303	√	AT5G20680	TBL16	37.7	1 239 520
Eucgr.A02463	√	AT5G06700	TBR	14.6	894 712
Eucgr.D01711	√	AT4G11090	TBL23	18.1	800 204
Eucgr.H04947	√	AT1G48880	TBL7	56.7	758 736
Eucgr.B03360		AT1G70230	TBL27	14.4	611 037
Eucgr.H01509	√	AT5G19160	TBL11	37.4	535 593
Eucgr.C00073	√	AT5G20590	TBL5	25.0	516 855
Eucgr.I00187	√	AT5G20680	TBL16	23.3	430 990
Eucgr.A00614		AT5G01620	TBL35	12.1	404 156
Eucgr.K00144	√	AT5G51640	TBL17,YLS7	19.2	392 996
Eucgr.B01121	√	AT3G06080	TBL10	11.2	367 683
Eucgr.A02628		AT3G11570	TBL8	100.0	306 559
Eucgr.I02119		AT5G15900	TBL19	10.4	287 311
Eucgr.F03235		AT1G29050	TBL38	58.7	271 226
Eucgr.B02240		AT5G58600	PMR5,TBL44	6.4	247 832
Eucgr.L01011		AT5G15900	TBL19	22.7	227 001
Eucgr.G01129	√	AT2G14530	TBL13	24.0	224 348
Eucgr.I02565	√	AT5G64470	TBL12	17.9	224 338
Eucgr.E02713	√	AT1G29050	TBL38	10.7	184 713
Eucgr.K02955	√	AT2G38320	TBL34	46.2	173 202
Eucgr.B03636	√	AT5G06700	TBR	12.1	169 615
Eucgr.K03547	√	AT3G62390	TBL6	13.5	150 346
Eucgr.A01900	√	AT2G42570	TBL39	2.4	115 506
Eucgr.H03574		AT5G19160	TBL11	59.1	106 346
Eucgr.I01832		AT3G28150	TBL22	14.2	98 310
Eucgr.J01893		AT5G58600	PMR5,TBL44	2.2	92 815
Eucgr.F01363		AT3G14850	TBL41	21.1	90 166
Eucgr.H02546		AT1G60790	TBL2	20.7	26 499
Eucgr.K01184		AT2G30900	TBL43	11.1	15 165
Eucgr.B03363		AT1G70230	TBL27	2.0	12 014
Eucgr.F01364		AT1G78710	TBL42	5.5	2 847
Eucgr.A01950		AT1G29050	TBL38	37.8	1 873
Eucgr.K01183		AT2G30900	TBL43	3.0	747
Eucgr.K02957		AT2G38320	TBL34	0.0	232
Eucgr.A01898		AT2G38320	TBL34	0.0	0
Eucgr.I02110		AT5G15900	TBL19	0.0	0
Eucgr.K02575		AT5G06230	TBL9	0.0	0
Eucgr.A01902		AT2G31110	TBL40	0.0	0
Eucgr.I02116		AT5G15900	TBL19	0.0	0
Eucgr.L01899		AT5G15900	TBL19	0.0	0
Eucgr.L01896		AT5G15900	TBL19	0.0	0
Eucgr.K02956		AT2G38320	TBL34	0.0	0
Eucgr.K02958		AT2G38320	TBL34	0.0	0
Eucgr.I02109		AT5G15900	TBL19	0.0	0
Eucgr.J00171		AT2G40320	TBL33	0.0	0
Eucgr.L03073		AT2G38320	TBL34	0.0	0
Eucgr.A01903		AT3G12060	TBL1	0.0	0

*Relative expression in developing secondary xylem. RNA-seq data obtained from EucGenIE [2].

†Absolute expression in developing secondary xylem. RNA-seq data obtained from EucGenIE [2].

Table S6. H3K4me3 enrichment of *E. grandis* genes involved in lignin biosynthesis. Gene lists and tandem duplication gene lists obtained from Myburg *et al.* [3].

Gene ID	H3K4me3-enriched?	*Arabidopsis* BLAST hit	*Arabidopsis* gene name	Relative expression (%)*	Absolute expression (FPKM)†	Tandem duplication?
**PAL**						
Eucgr.J01079		AT3G53260	PAL2	45.4	6 744 160	
Eucgr.G02848		AT2G37040	PAL1	80.0	2 371 820	√
Eucgr.J00907		AT3G53260	PAL2	6.8	465 141	
Eucgr.G02852		AT3G53260	PAL2	57.8	383 305	√
Eucgr.G02850		AT3G53260	PAL2	48.3	199 783	√
Eucgr.G02851		AT3G53260	PAL2	47.2	72 363	√
Eucgr.G02849		AT3G53260	PAL2	23.6	24 912	√
Eucgr.C03570		AT3G53260	PAL2	1.6	16 124	
Eucgr.A01144		AT2G37040	PAL1	7.7	742	
**C4H**						
Eucgr.J01844	√	AT2G30490	C4H	53.7	18 372 167	
Eucgr.C00065		AT2G30490	C4H	87.0	2 574 721	
**4CL**						
Eucgr.C02284	√	AT1G51680	4CL1	57.2	12 572 167	
Eucgr.K00087		AT1G65060	4CL3	3.8	324 693	
Eucgr.B03943		AT4G05160		4.0	242 345	√
Eucgr.F03543		AT1G20510	OPCL1	3.8	224 078	
Eucgr.B03502		AT1G62940	ACOS5	6.9	82 751	
Eucgr.K02929		AT4G05160		4.7	57 076	√
Eucgr.D02624		AT4G19010		0.8	31 170	
Eucgr.B03942	√	AT4G05160		1.9	14 908	√
Eucgr.B03468		AT5G63380		2.8	2 995	
Eucgr.B00135		AT5G63380		1.2	505	√
Eucgr.K02927		AT4G05160		1.8	472	√
Eucgr.G02758		AT1G20510	OPCL1	0.0	0	
Eucgr.G02879		AT1G20510	OPCL1	0.0	0	
**HCT**						
Eucgr.J03126	√	AT5G48930	HCT	58.2	6 624 317	
Eucgr.F03978	√	AT5G48930	HCT	56.6	2 639 093	√
Eucgr.F03972		AT5G48930	HCT	3.2	7 372	√
Eucgr.F03973		AT5G48930	HCT	0.3	3 329	√
Eucgr.F03974		AT5G48930	HCT	0.2	823	√
**C3H**						
Eucgr.A02190		AT2G40890	CYP98A3	70.4	10 346 810	√
Eucgr.G03199		AT2G40890	CYP98A3	79.5	158 734	
Eucgr.A02188		AT2G40890	CYP98A3	6.0	48 760	√
Eucgr.A02185		AT2G40890	CYP98A3	0.7	23 518	√
**CCoAOMT**						
Eucgr.G01417	√	AT4G34050	CCoAOMT1	69.9	32 118 967	
Eucgr.I01134	√	AT4G34050	CCoAOMT1	66.3	16 420 333	
Eucgr.F04260		AT3G62000		5.2	65 545	
Eucgr.H04648		AT1G67980		1.6	44 468	√
Eucgr.H04646		AT1G67980		0.1	5 880	√
Eucgr.H04650		AT1G67980		0.6	5 313	√
Eucgr.C03674		AT4G26220		2.2	1 119	√
Eucgr.C03680		AT4G26220		0.8	1 108	√
Eucgr.B02687		AT1G67980		0.0	0	
Eucgr.H04643		AT1G67980		0.0	0	√
Eucgr.H04644		AT1G67980		0.0	0	√
Eucgr.C00924		AT4G26220		0.0	0	√
Eucgr.C00925		AT4G26220		0.0	0	√
Eucgr.C03667		AT4G26220		0.0	0	√
Eucgr.C03668		AT4G26220		0.0	0	√
Eucgr.C03684		AT4G26220		0.0	0	√
Eucgr.C03939		AT4G26220		0.0	0	√
**F5H**						
Eucgr.J02393		AT4G36220	CYP84A1,FAH1	93.0	22 535 133	
Eucgr.I02371		AT4G36220	CYP84A1,FAH1	0.1	1 385	

COMT

Eucgr.A01397	AT5G54160	OMT1	82.4	49 992 600	√
Eucgr.K00951	AT5G54160	OMT1	76.7	7 964 113	√
Eucgr.F02623	AT5G54160	OMT1	64.1	360 953	√
Eucgr.K00955	AT4G35150		58.7	286 001	√
Eucgr.F03794	AT5G54160	OMT1	4.2	160 802	
Eucgr.E03875	AT5G54160	OMT1	0.0	77 382	√
Eucgr.A01797	AT4G35160		3.6	49 085	√
Eucgr.K00957	AT5G54160	OMT1	9.7	38 356	√
Eucgr.B01744	AT5G54160	OMT1	0.0	37 768	√
Eucgr.B01747	AT5G54160	OMT1	0.0	34 309	√
Eucgr.A01600	AT3G53140		2.4	30 305	
Eucgr.E03874	AT5G54160	OMT1	0.0	27 783	√
Eucgr.A01796	AT4G35160		0.4	21 308	√
Eucgr.A01846	AT4G35160		2.4	19 679	
Eucgr.F02624	AT5G54160	OMT1	2.1	14 097	√
Eucgr.H03926	AT5G54160	OMT1	0.2	13 143	√
Eucgr.E01092	AT5G54160	OMT1	5.7	10 763	
Eucgr.A01795	AT4G35160		13.7	10 104	√
Eucgr.K00956	AT5G54160	OMT1	16.4	8 380	√
Eucgr.E03148	AT5G54160	OMT1	3.7	7 030	√
Eucgr.H03920	AT5G54160	OMT1	0.3	4 248	√
Eucgr.K01696	AT5G54160	OMT1	14.3	4 182	√
Eucgr.H00354	AT1G51990		0.1	3 581	√
Eucgr.H03924	AT5G54160	OMT1	0.2	2 951	√
Eucgr.A01392	AT5G54160	OMT1	0.0	1 752	√
Eucgr.E03877	AT5G54160	OMT1	0.0	1 716	√
Eucgr.E03146	AT5G54160	OMT1	3.5	1 692	√
Eucgr.H03922	AT5G54160	OMT1	0.4	1 337	√
Eucgr.A01877	AT4G35150		4.6	717	√
Eucgr.H00356	AT5G54160	OMT1	0.0	705	√
Eucgr.A01884	AT4G35160		1.8	676	√
Eucgr.E03341	AT1G77520		0.6	539	√
Eucgr.A01395	AT5G54160	OMT1	0.0	526	√
Eucgr.E03339	AT5G54160	OMT1	0.7	464	√
Eucgr.A01865	AT5G54160	OMT1	12.3	442	√
Eucgr.H00348	AT5G54160	OMT1	4.2	418	√
Eucgr.A02870	AT5G54160	OMT1	0.0	344	
Eucgr.H00353	AT5G54160	OMT1	0.0	343	√
Eucgr.G00020	AT5G54160	OMT1	0.0	281	√
Eucgr.A01881	AT4G35160		1.2	280	√
Eucgr.G00017	AT5G54160	OMT1	0.0	272	√
Eucgr.A01874	AT4G35150		0.0	0	√
Eucgr.A01876	AT4G35150		0.0	0	√
Eucgr.A01878	AT4G35150		0.0	0	√
Eucgr.A01880	AT4G35150		0.0	0	√
Eucgr.A01863	AT4G35160		0.0	0	√
Eucgr.A01873	AT4G35160		0.0	0	√
Eucgr.A01875	AT4G35160		0.0	0	√
Eucgr.G01808	AT4G35160		0.0	0	√
Eucgr.G01810	AT4G35160		0.0	0	√
Eucgr.A00759	AT5G54160	OMT1	0.0	0	
Eucgr.A01389	AT5G54160	OMT1	0.0	0	√
Eucgr.A01394	AT5G54160	OMT1	0.0	0	√
Eucgr.A01867	AT5G54160	OMT1	0.0	0	√
Eucgr.F02625	AT5G54160	OMT1	0.0	0	√
Eucgr.H00347	AT5G54160	OMT1	0.0	0	√
Eucgr.H00349	AT5G54160	OMT1	0.0	0	√
Eucgr.H00350	AT5G54160	OMT1	0.0	0	√
Eucgr.H00351	AT5G54160	OMT1	0.0	0	√
Eucgr.H00352	AT5G54160	OMT1	0.0	0	√
Eucgr.I02810	AT5G54160	OMT1	0.0	0	
Eucgr.K00041	AT5G54160	OMT1	0.0	0	
Eucgr.K00449	AT5G54160	OMT1	0.0	0	
Eucgr.K00949	AT5G54160	OMT1	0.0	0	√
Eucgr.K00950	AT5G54160	OMT1	0.0	0	√

Eucgr.K00953		AT5G54160	OMT1	0.0	0	√
Eucgr.K00954		AT5G54160	OMT1	0.0	0	√
**CCR**						
Eucgr.J03114	√	AT1G15950	CCR1	41.2	5 899 387	
Eucgr.B02222		AT5G58490		30.7	3 293 937	
Eucgr.C01240	√	AT2G23910		16.8	598 960	
Eucgr.G00052		AT2G02400		14.9	303 772	
Eucgr.F03605	√	AT2G33590		4.9	197 975	√
Eucgr.I01783	√	AT5G14700		9.5	152 750	
Eucgr.G02325		AT1G68540		0.3	5 870	
Eucgr.I01552		AT4G35420	DRL1	1.2	4 890	
Eucgr.F03954		AT1G15950	CCR1	0.0	0	
**CAD**						
Eucgr.E01119	√	AT1G72680	CAD1	18.8	2 092 140	√
Eucgr.E01117		AT1G72680	CAD1	20.9	1 482 223	√
Eucgr.G01350		AT3G19450	CAD4	40.8	543 344	
Eucgr.E01107	√	AT1G72680	CAD1	20.6	351 410	√
Eucgr.H04903		AT4G39330	CAD9	2.6	329 604	√
Eucgr.E01110		AT1G72680	CAD1	10.2	87 075	√
Eucgr.F01680		AT4G39330	CAD9	1.8	58 938	√
Eucgr.F01676		AT4G39330	CAD9	2.3	24 324	√
Eucgr.I00570		AT4G37980	CAD7	0.2	16 613	√
Eucgr.E01103		AT1G72680	CAD1	6.4	11 802	√
Eucgr.K01941		AT4G37990	CAD8	15.3	10 945	
Eucgr.F01679		AT4G39330	CAD9	0.7	4 997	√
Eucgr.F01678		AT4G39330	CAD9	0.8	3 910	√
Eucgr.E01105		AT1G72680	CAD1	1.8	3 848	√
Eucgr.G02223		AT4G39330	CAD9	2.8	3 809	
Eucgr.E01115		AT1G72680	CAD1	0.1	2 242	√
Eucgr.E01104		AT1G72680	CAD1	0.5	839	√
Eucgr.E01108		AT1G72680	CAD1	0.2	696	√
Eucgr.D00472		AT1G72680	CAD1	24.6	659	√
Eucgr.F01677		AT4G39330	CAD9	2.8	436	√
Eucgr.D01087		AT4G37980	CAD7	0.4	319	√
Eucgr.D00468		AT1G72680	CAD1	0.0	0	√
Eucgr.D00471		AT1G72680	CAD1	0.0	0	√
Eucgr.D00473		AT1G72680	CAD1	0.0	0	√
Eucgr.D01090		AT4G37980	CAD7	0.0	0	√
Eucgr.E02204		AT4G37980	CAD7	0.0	0	
Eucgr.E02310		AT4G37980	CAD7	0.0	0	√
Eucgr.E02319		AT4G37980	CAD7	0.0	0	√
Eucgr.E02559		AT4G37980	CAD7	0.0	0	√
Eucgr.E02570		AT4G37980	CAD7	0.0	0	√
Eucgr.E02580		AT4G37980	CAD7	0.0	0	√
Eucgr.H02411		AT4G37980	CAD7	0.0	0	√
Eucgr.H02434		AT4G37980	CAD7	0.0	0	√
Eucgr.D01088		AT4G37980	CAD7	0.0	0	√
Eucgr.D01089		AT4G37990	CAD8	0.0	0	√
Eucgr.H02433		AT4G37990	CAD8	0.0	0	√
Eucgr.I00571		AT4G37990	CAD8	0.0	0	√
Eucgr.I00572		AT4G37990	CAD8	0.0	0	√
Eucgr.I00573		AT4G37990	CAD8	0.0	0	√
Eucgr.D01091		AT4G39330	CAD9	0.0	0	√
Eucgr.H02412		AT4G39330	CAD9	0.0	0	√
Eucgr.H02414		AT4G39330	CAD9	0.0	0	√
Eucgr.H02415		AT4G39330	CAD9	0.0	0	√
Eucgr.H02431		AT4G39330	CAD9	0.0	0	√
Eucgr.D01086		AT4G39330	CAD9	0.0	0	√

*Relative expression in developing secondary xylem. RNA-seq data obtained from EucGenIE [2].
†Absolute expression in developing secondary xylem. RNA-seq data obtained from EucGenIE [2].

Table S7. List of primers used for qPCR analysis

Locus	Annotation	Primers	Amplicon length (bp)
scaffold_8:33,795,824-33,795,938	Eucgr.H02469	5'-GATCGAGAGTTCGGCGCATA-3'<br>5'-CATACGCCACTGCAGGCAAT-3'	134
scaffold_9:38,403,849-38,403,997	Eucgr.I02739	5'-CATGCTCATTCTCCGCATGT-3'<br>5'-GTCACTTTCACCCTTCCTCTC-3'	149
scaffold_8:66,987,046-66,987,137	Eucgr.H04673	5'-CTGGCGTTGGATACAATGTT-3'<br>5'-TGGTGCTAAGAAGGTTGTCA-3'	111
scaffold_1:21,274,282-21,274,366	Eucgr.A01324	5'-GTGCAAATGACTCCCAAGAA-3'<br>5'-GATCAGATCACCGAGGACAA-3'	104
scaffold_3:5,079,522-5,079,592	Eucgr.C00246	5'-TTGAGCGTTATCGTCATCCT-3'<br>5'-GAGCAGAACAAGCACAAGTA-3'	90
scaffold_4:8,617,282-8,617,403	Eucgr.D00476	5'-GTTGCTCAGTCATGGCATTC-3'<br>5'-TAGGGCCTAAGACCAAACAC-3'	141
scaffold_10:3,764,427..3,764,590	Eucgr.J00384	5'-TAGCCGTGCAAGAGCCTCAT-3'<br>5'-TTCATCATCACCGCCATCGC-3'	183
scaffold_9:18,069,028..18,069,166	Eucgr.I00880	5'-GCACAATTCTGCTCCGATGA-3'<br>5'-AGTGCAAGGCTGTGAATCTC-3'	158
scaffold_10:38,339,144..38,339,239	Eucgr.J03126	5'-ATGGCCGCATTGAGATTGAC-3'<br>5'-AGCTTCCGAAGCTCCAATGT-3'	115
scaffold_5:11,792,297..11,792,357	Eucgr.E01107	5'-ATTCAGCGCATACACAACAA-3'<br>5'-GAAGTGTTCATGCGAGACAG-3'	80
scaffold_5:11,291,880..11,292,032	Eucgr.E01053	5'-TCACGTCCAAGTCGATCTTC-3’<br>5'-GCTGAGCATACAGCTCGTTA-3'	172
scaffold_11:13,335,435..13,335,577	Eucgr.K01061	5'-ACCAAGACGACTATGCTAGAA-3'<br>5'-CACCGCCATCCAACAATAA-3'	161
scaffold_4:30,695,364..30,695,525	Eucgr.D01671	5'-GCAGCGTCCTGGATCAGATA-3'<br>5'-AATGTCCTCAAGCCGGTCTC-3'	181
scaffold_10:29,822,933..29,823,118	Eucgr.J02393	5'-GCGATCAAGATGTCGATACC-3'<br>5’-GGTGAACCGAGCAAGATTAG-3’	205
scaffold_1:22,485,326..22,485,482	Eucgr.A01397	5'-TGGTCCGCGTAATATGATGG-3'<br>5'-TGGTGGTGAGAATTGCAGAG-3'	176
scaffold_11:11,514,296..11,514,420	Eucgr.K00951	5'-ACTGCATCAGCATGTGGTAT-3'<br>5'-AGTGGCCGAGATCATTAAGT-3'	144
scaffold_8:58,195,203..58,195,428	Intergenic (Negative 1)	5'-CTCGACTGTGAAGAGCTATC-3'<br>5'-CAGAGTAGCCATTCTCAAGG-3'	245
scaffold_11:13,340,627..13,340,821	Intergenic (Negative 2)	5'-ATATGGTGTCACATTGCATCAG-3'<br>5'-ATCGGCTAATGTCTCAATCAAG-3'	216

References

1. Dai X, Zhao PX: **psRNATarget: A plant small RNA target analysis server**. *Nucleic Acids Research* 2011, **39.suppl 2**:W155-W159.
2. Hefer C, Mizrachi E, Joubert F, Myburg A: **The *Eucalyptus* genome integrative explorer (EucGenIE): a resource for *Eucalyptus* genomics and transcriptomics**. *BMC Proceedings* 2011, **5**(Suppl 7):O49.

3. Myburg AA, Grattapaglia D, Tuskan GA, Hellsten U, Hayes RD, Grimwood J, Jenkins J, Lindquist E, Tice H, Bauer D *et al*: **The genome of *Eucalyptus grandis* - a global tree for fiber and energy**. *Nature* 2014, **510**:356-362.
